# Supplementary material for: Variable crab camouflage patterns defeat search image formation
Source: Commun Biol. 2021 Mar 5;4:287. doi: 10.1038/s42003-021-01817-8 (PMC7935895; doi:10.1038/s42003-021-01817-8)
Supplement: Supplementary file 2 — Description of Additional Supplementary Files [file 42003_2021_1817_MOESM2_ESM.pdf]

Description of Additional Supplementary Files

File name: Supplementary Data

Description:

Supplementary Data 1: Metrics for crab appearance analysis

Supplementary Data 2: This CSV file contains all the raw data used by the R code to perform all statistics.

Supplementary Data 3: This is an rMarkdown document containing the code and output from our analysis.
