# Supplementary material for: Variable crab camouflage patterns defeat search image formation
Source: Commun Biol. 2021 Mar 5;4:287. doi: 10.1038/s42003-021-01817-8 (PMC7935895; doi:10.1038/s42003-021-01817-8)
Supplement: Supplementary file 3 — Supplementary Data [file 42003_2021_1817_MOESM3_ESM.zip › Supplementary Data 3.pdf]

Crab Search Image

Jolyon Troscianko

Analysis of crab camouflage and search image effects

```
# setwd("/home/jolyon/Documents/Work/Camouflage Learning/Crab Game/Stats")

library(rmarkdown)
library(gtools)
library(coxme)
```

```
## Loading required package: survival
```

```
## Loading required package: bdsmatrix
```

```
##
## Attaching package: 'bdsmatrix'
```

```
## The following object is masked from 'package:base':
##
##     backsolve
```

```
library(ggplot2)
library(gridExtra)
library(lme4)
```

```
## Loading required package: Matrix
```

```
library(LMERConvenienceFunctions)

data <- read.csv("CrabCamoResults.csv")

# str(data)
data$bgID <- factor(data$bgID)
data$crabID <- factor(data$crabID)
data$sessionID <- factor(data$sessionID)

data$hitOrTimeout <- ifelse(data$hit == 1, 1, ifelse(data$time=="timeout",1,0))
data <- subset(data, hitOrTimeout == 1)

# calculate number of encounters with each crab
seqCount <- 0
crabCounter <- 1 # for some reason crabSeq sometimes starts with a zero, and somet
imes a 1, so is fixed below.
for(i in 1:(length(data$crabSeq)-1)){
  data$crabSeq[i] <- crabCounter
    seqCount <- seqCount + 1
    data$encounters[i] <- seqCount
    if(data$crabID[i] != data$crabID[i+1] | data$sessionID[i] != data$sessionID[i+
1])
        seqCount <- 0

    if(data$crabID[i] != data$crabID[i+1])
      crabCounter <- crabCounter + 1

    if(data$sessionID[i] != data$sessionID[i+1])
      crabCounter <- 1

}
# last row:
if(data$crabID[length(data$crabSeq)] == data$crabID[length(data$crabSeq)-1]){
  data$crabSeq[length(data$crabSeq)] <- data$crabSeq[length(data$crabSeq)-1]
  data$encounters[length(data$crabSeq)] <- data$encounters[length(data$crabSeq)-1]
+1
} else{
  data$crabSeq[length(data$crabSeq)] <- data$crabSeq[length(data$crabSeq)-1] +1
  data$encounters[length(data$crabSeq)] <- 1
}

# hist(data$encounters)
# max(data$encounters)



# check for duplicate session data (this happens when people click the 'submit' bu
tton repeatedly
data$duplicate <- 0
dupl <-0
for(i in 1:length(data$crabSeq))
    if(data$slide[i] == 1)
        for(j in (i+1):length(data$crabSeq)){
            if(data$slide[j] == 1 && data$duplicate[j] == 0){
                dupl <- 0
```

```
                if(data$cTime[j] == data$cTime[i]){
                    if(data$xCoords[i] == data$xCoords[j] && data$yCoords[i] == da
ta$yCoords[j]){
                        # print(j)
                        dupl <- 1
                     }
                }
            }
            if(dupl == 1)
                    data$duplicate[j] <- i
        }

# nlevels(factor(data$sessionID))

data <- subset(data, duplicate == 0)

nlevels(factor(data$sessionID))
```

```
## [1] 1751
```

```
# final number of unique game plays: 1751

#
# hist(as.numeric(data$crabID))
# min(as.numeric(data$crabID))
# max(as.numeric(data$crabID))
# hist(as.numeric(data$bgID))
# crabs and backgrounds nice and evenly distributed
# hist(data$cTime, breaks=100)
# hist(log(data$cTime), breaks=100)

# -------------------------Determine which metrics best predict performance
---------------------------
# -------------------------Distuption-------------------------------

# d1 <- coxme(Surv(cTime, hit) ~ screenScale + playedBefore + poly(crab_circular_f
it_centre_x,2) + poly(crab_circular_fit_centre_y,2) + L_GabRat_sig2.0 + crab_area
+ (1|sessionID), data)
# summary(d1) # L_GabRat_sig2.0 z=-58.04 p=0

d2 <- coxme(Surv(cTime, hit) ~ screenScale + playedBefore + poly(crab_circular_fit
_centre_x,2) + poly(crab_circular_fit_centre_y,2) + L_GabRat_sig3.0 + crab_area +
(1|sessionID), data)
summary(d2) # L_GabRat_sig3.0 z=-58.16 p=0
```

```
## Cox mixed-effects model fit by maximum likelihood
##   Data: data
##   events, n = 40354, 42023
##   Iterations= 13 73
##                     NULL Integrated    Fitted
## Log-likelihood -394639.4  -386023.7 -383231.2
##
##                       Chisq      df p      AIC      BIC
## Integrated loglik 17231.28    9.00 0 17213.28 17135.84
##  Penalized loglik 22816.31 1578.21 0 19659.88  6078.66
##
## Model:  Surv(cTime, hit) ~ screenScale + playedBefore + poly(crab_circular_fit_
centre_x,      2) + poly(crab_circular_fit_centre_y, 2) + L_GabRat_sig3.0 +      c
rab_area + (1 | sessionID)
## Fixed coefficients
##                                               coef    exp(coef)
## screenScale                            3.946409e-01 1.483851e+00
## playedBeforey                          4.714141e-01 1.602258e+00
## poly(crab_circular_fit_centre_x, 2)1 -1.393195e+01 8.900860e-07
## poly(crab_circular_fit_centre_x, 2)2 -4.118473e+01 1.299272e-18
## poly(crab_circular_fit_centre_y, 2)1 -1.056839e+01 2.571611e-05
## poly(crab_circular_fit_centre_y, 2)2 -3.183265e+01 1.497116e-14
## L_GabRat_sig3.0                       -7.030083e+00 8.848580e-04
## crab_area                              1.859851e-05 1.000019e+00
##                                          se(coef)      z       p
## screenScale                           5.452840e-02   7.24 4.6e-13
## playedBeforey                         3.162943e-02  14.90 0.0e+00
## poly(crab_circular_fit_centre_x, 2)1 1.062961e+00 -13.11 0.0e+00
## poly(crab_circular_fit_centre_x, 2)2 1.087963e+00 -37.85 0.0e+00
## poly(crab_circular_fit_centre_y, 2)1 1.067424e+00  -9.90 0.0e+00
## poly(crab_circular_fit_centre_y, 2)2 1.098197e+00 -28.99 0.0e+00
## L_GabRat_sig3.0                       1.208764e-01 -58.16 0.0e+00
## crab_area                             4.178056e-07  44.51 0.0e+00
##
## Random effects
##  Group     Variable  Std Dev   Variance
##  sessionID Intercept 0.6178442 0.3817315
```

```
# d3 <- coxme(Surv(cTime, hit) ~ screenScale + playedBefore + poly(crab_circular_f
it_centre_x,2) + poly(crab_circular_fit_centre_y,2) + L_GabRat_sig4.0 + crab_area
+ (1|sessionID), data)
# summary(d3) # L_GabRat_sig4.0 z=-57.51 p=0
#
# d4 <- coxme(Surv(cTime, hit) ~ screenScale + playedBefore + poly(crab_circular_f
it_centre_x,2) + poly(crab_circular_fit_centre_y,2) + A_GabRat_sig2.0 + crab_area
+ (1|sessionID), data)
# summary(d4) # A_GabRat_sig2.0 z=-33.79 p=0
#
# d5 <- coxme(Surv(cTime, hit) ~ screenScale + playedBefore + poly(crab_circular_f
it_centre_x,2) + poly(crab_circular_fit_centre_y,2) + A_GabRat_sig3.0 + crab_area
+ (1|sessionID), data)
# summary(d5) # A_GabRat_sig3.0 z=-34.87 p=0
#
d6 <- coxme(Surv(cTime, hit) ~ screenScale + playedBefore + poly(crab_circular_fit
_centre_x,2) + poly(crab_circular_fit_centre_y,2) + A_GabRat_sig4.0 + crab_area +
(1|sessionID), data)
summary(d6) # A_GabRat_sig4.0 z=-35.07 p=0
```

```
## Cox mixed-effects model fit by maximum likelihood
##   Data: data
##   events, n = 40354, 42023
##   Iterations= 13 72
##                       NULL Integrated    Fitted
## Log-likelihood -394639.4  -387091.8 -384356.7
##
##                        Chisq      df p      AIC      BIC
## Integrated loglik 15095.07    9.00 0 15077.07 14999.62
##  Penalized loglik 20565.33 1568.27 0 17428.80  3933.17
##
## Model:  Surv(cTime, hit) ~ screenScale + playedBefore + poly(crab_circular_fit_
centre_x,      2) + poly(crab_circular_fit_centre_y, 2) + A_GabRat_sig4.0 +      c
rab_area + (1 | sessionID)
## Fixed coefficients
##                                               coef    exp(coef)
## screenScale                            3.478768e-01 1.416058e+00
## playedBeforey                          4.520270e-01 1.571494e+00
## poly(crab_circular_fit_centre_x, 2)1 -1.142804e+01 1.088590e-05
## poly(crab_circular_fit_centre_x, 2)2 -3.918850e+01 9.564224e-18
## poly(crab_circular_fit_centre_y, 2)1 -9.722100e+00 5.994401e-05
## poly(crab_circular_fit_centre_y, 2)2 -2.989148e+01 1.043024e-13
## A_GabRat_sig4.0                       -3.166618e+00 4.214589e-02
## crab_area                              1.933006e-05 1.000019e+00
##                                          se(coef)      z       p
## screenScale                           5.425216e-02   6.41 1.4e-10
## playedBeforey                         3.084777e-02  14.65 0.0e+00
## poly(crab_circular_fit_centre_x, 2)1 1.064231e+00 -10.74 0.0e+00
## poly(crab_circular_fit_centre_x, 2)2 1.086289e+00 -36.08 0.0e+00
## poly(crab_circular_fit_centre_y, 2)1 1.066931e+00  -9.11 0.0e+00
## poly(crab_circular_fit_centre_y, 2)2 1.094629e+00 -27.31 0.0e+00
## A_GabRat_sig4.0                       9.029679e-02 -35.07 0.0e+00
## crab_area                             4.256436e-07  45.41 0.0e+00
##
## Random effects
##  Group     Variable  Std Dev   Variance
##  sessionID Intercept 0.5995484 0.3594583
```

```
#
# d7 <- coxme(Surv(cTime, hit) ~ screenScale + playedBefore + poly(crab_circular_f
it_centre_x,2) + poly(crab_circular_fit_centre_y,2) + B_GabRat_sig2.0 + crab_area
+ (1|sessionID), data)
# summary(d7) # B_GabRat_sig2.0 z=-28.10 p=0
#
# d8 <- coxme(Surv(cTime, hit) ~ screenScale + playedBefore + poly(crab_circular_f
it_centre_x,2) + poly(crab_circular_fit_centre_y,2) + B_GabRat_sig3.0 + crab_area
+ (1|sessionID), data)
# summary(d8) # B_GabRat_sig3.0 z=-29.48 p=0
#
# d9 <- coxme(Surv(cTime, hit) ~ screenScale + playedBefore + poly(crab_circular_f
it_centre_x,2) + poly(crab_circular_fit_centre_y,2) + B_GabRat_sig4.0 + crab_area
+ (1|sessionID), data)
# summary(d9) # B_GabRat_sig4.0 z=-30.16 p=0

# -------------------------Pattern---------------------------------

p1 <- coxme(Surv(cTime, hit) ~ screenScale + playedBefore + poly(crab_circular_fit
_centre_x,2) + poly(crab_circular_fit_centre_y,2) + crab.surr_DoGdiff + crab_area
+ (1|sessionID), data)
summary(p1) # crab.surr_DoGdiff z=8.41 p=0
```

```
## Cox mixed-effects model fit by maximum likelihood
##   Data: data
##   events, n = 40354, 42023
##   Iterations= 13 72
##                     NULL Integrated    Fitted
## Log-likelihood -394639.4  -387665.6 -384939.4
##
##                       Chisq      df p      AIC      BIC
## Integrated loglik 13947.55    9.00 0 13929.55 13852.10
##  Penalized loglik 19399.90 1566.62 0 16266.66  2785.21
##
## Model:  Surv(cTime, hit) ~ screenScale + playedBefore + poly(crab_circular_fit_
centre_x,      2) + poly(crab_circular_fit_centre_y, 2) + crab.surr_DoGdiff +
crab_area + (1 | sessionID)
## Fixed coefficients
##                                              coef    exp(coef)
## screenScale                          3.421043e-01 1.407907e+00
## playedBeforey                        4.383604e-01 1.550164e+00
## poly(crab_circular_fit_centre_x, 2)1 -1.342752e+01 1.474015e-06
## poly(crab_circular_fit_centre_x, 2)2 -3.762282e+01 4.577375e-17
## poly(crab_circular_fit_centre_y, 2)1 -1.026206e+01 3.493364e-05
## poly(crab_circular_fit_centre_y, 2)2 -2.916839e+01 2.149465e-13
## crab.surr_DoGdiff                    1.656001e-02 1.016698e+00
## crab_area                            2.411314e-05 1.000024e+00
##                                          se(coef)      z       p
## screenScale                          5.435061e-02   6.29 3.1e-10
## playedBeforey                        3.073061e-02  14.26 0.0e+00
## poly(crab_circular_fit_centre_x, 2)1 1.062342e+00 -12.64 0.0e+00
## poly(crab_circular_fit_centre_x, 2)2 1.084379e+00 -34.70 0.0e+00
## poly(crab_circular_fit_centre_y, 2)1 1.066617e+00  -9.62 0.0e+00
## poly(crab_circular_fit_centre_y, 2)2 1.094872e+00 -26.64 0.0e+00
## crab.surr_DoGdiff                    1.970081e-03   8.41 0.0e+00
## crab_area                            4.070158e-07  59.24 0.0e+00
##
## Random effects
##  Group     Variable  Std Dev   Variance
##  sessionID Intercept 0.5966327 0.3559706
```

```r
# p2 <- coxme(Surv(cTime, hit) ~ screenScale + playedBefore + poly(crab_circular_f
it_centre_x,2) + poly(crab_circular_fit_centre_y,2) + crab.bg_DoGdiff + crab_area
+ (1|sessionID), data)
# summary(p2) # crab.bg_DoGdiff z=-0.12 p=0.9

# Best Pattern = crab.surr_DoGdiff

# ------------------------Luminance-------------------------

# l1 <- coxme(Surv(cTime, hit) ~ screenScale + playedBefore + poly(crab_circular_f
it_centre_x,2) + poly(crab_circular_fit_centre_y,2) + poly(crab_L_mean,2) + crab_a
rea + (1|sessionID), data)
# summary(l1) # crab_L_mean z=-24.58 & 26.60 p=0
#
# l2 <- coxme(Surv(cTime, hit) ~ screenScale + playedBefore + poly(crab_circular_f
it_centre_x,2) + poly(crab_circular_fit_centre_y,2) + crab.surr_Euclidean_L_dist +
crab_area + (1|sessionID), data)
# summary(l2) # crab.surr_Euclidean_L_dist z=54.71 p=0
#
# l3 <- coxme(Surv(cTime, hit) ~ screenScale + playedBefore + poly(crab_circular_f
it_centre_x,2) + poly(crab_circular_fit_centre_y,2) + crab.bg_Euclidean_L_dist + c
rab_area + (1|sessionID), data)
# summary(l3) # crab.bg_Euclidean_L_dist z=49.00 p=0

l4 <- coxme(Surv(cTime, hit) ~ screenScale + playedBefore + poly(crab_circular_fit
_centre_x,2) + poly(crab_circular_fit_centre_y,2) + surr_L_diff + crab_area + (1|s
essionID), data)
summary(l4) # surr_L_diff z=56.20 p=0
```

```
## Cox mixed-effects model fit by maximum likelihood
##   Data: data
##   events, n = 40354, 42023
##   Iterations= 13 73
##                     NULL Integrated  Fitted
## Log-likelihood -394639.4  -386142.7 -383360
##
##                      Chisq      df p      AIC      BIC
## Integrated loglik 16993.27    9.00 0 16975.27 16897.83
##  Penalized loglik 22558.77 1576.54 0 19405.70  5838.90
##
## Model:  Surv(cTime, hit) ~ screenScale + playedBefore + poly(crab_circular_fit_
centre_x,      2) + poly(crab_circular_fit_centre_y, 2) + surr_L_diff +      crab_
area + (1 | sessionID)
## Fixed coefficients
##                                               coef    exp(coef)
## screenScale                           3.671932e-01 1.443677e+00
## playedBeforey                         4.722439e-01 1.603588e+00
## poly(crab_circular_fit_centre_x, 2)1 -1.395800e+01 8.671976e-07
## poly(crab_circular_fit_centre_x, 2)2 -4.023632e+01 3.354205e-18
## poly(crab_circular_fit_centre_y, 2)1 -1.108365e+01 1.536151e-05
## poly(crab_circular_fit_centre_y, 2)2 -3.146402e+01 2.164457e-14
## surr_L_diff                           8.209534e-01 2.272666e+00
## crab_area                             2.232971e-05 1.000022e+00
##                                          se(coef)      z       p
## screenScale                          5.482340e-02   6.70 2.1e-11
## playedBeforey                        3.146800e-02  15.01 0.0e+00
## poly(crab_circular_fit_centre_x, 2)1 1.063003e+00 -13.13 0.0e+00
## poly(crab_circular_fit_centre_x, 2)2 1.087204e+00 -37.01 0.0e+00
## poly(crab_circular_fit_centre_y, 2)1 1.067609e+00 -10.38 0.0e+00
## poly(crab_circular_fit_centre_y, 2)2 1.096430e+00 -28.70 0.0e+00
## surr_L_diff                          1.460696e-02  56.20 0.0e+00
## crab_area                            4.093305e-07  54.55 0.0e+00
##
## Random effects
##  Group     Variable  Std Dev   Variance
##  sessionID Intercept 0.6146692 0.3778182
```

```
#
# l5 <- coxme(Surv(cTime, hit) ~ screenScale + playedBefore + poly(crab_circular_f
it_centre_x,2) + poly(crab_circular_fit_centre_y,2) + bg_L_diff + crab_area + (1|s
essionID), data)
# summary(l5) # bg_L_diff z=43.36 p=0
#
# l6 <- coxme(Surv(cTime, hit) ~ screenScale + playedBefore + poly(crab_circular_f
it_centre_x,2) + poly(crab_circular_fit_centre_y,2) + crab_L_sd + crab_area + (1|s
essionID), data)
# summary(l6) # crab_L_sd z=21.28 p=0

# Best Luminance = surr_L_diff

# -------------------------Colour------------------------------
#
# c1 <- coxme(Surv(cTime, hit) ~ screenScale + playedBefore + poly(crab_circular_f
it_centre_x,2) + poly(crab_circular_fit_centre_y,2) + surr_AB_diff + crab_area +
(1|sessionID), data)
# summary(c1) # surr_AB_diff z=36.11 p=0
#
# c2 <- coxme(Surv(cTime, hit) ~ screenScale + playedBefore + poly(crab_circular_f
it_centre_x,2) + poly(crab_circular_fit_centre_y,2) + bg_AB_diff + crab_area + (1|
sessionID), data)
# summary(c2) # bg_AB_diff z=26.81 p=0
#
# c3 <- coxme(Surv(cTime, hit) ~ screenScale + playedBefore + poly(crab_circular_f
it_centre_x,2) + poly(crab_circular_fit_centre_y,2) + crab.surr_Euclidean_AB_dist
+ crab_area + (1|sessionID), data)
# summary(c3) # crab.surr_Euclidean_AB_dist z=33.06 p=0

c4 <- coxme(Surv(cTime, hit) ~ screenScale + playedBefore + poly(crab_circular_fit
_centre_x,2) + poly(crab_circular_fit_centre_y,2) + crab.bg_Euclidean_AB_dist + cr
ab_area + (1|sessionID), data)
summary(c4) # crab.bg_Euclidean_AB_dist z=38.36 p=0
```

```
## Cox mixed-effects model fit by maximum likelihood
##   Data: data
##   events, n = 40354, 42023
##   Iterations= 13 72
##                       NULL Integrated    Fitted
## Log-likelihood -394639.4  -386980.8 -384248.5
##
##                        Chisq      df p      AIC      BIC
## Integrated loglik 15317.13    9.00 0 15299.13 15221.68
##  Penalized loglik 20781.78 1567.68 0 17646.41  4155.81
##
## Model:  Surv(cTime, hit) ~ screenScale + playedBefore + poly(crab_circular_fit_
centre_x,      2) + poly(crab_circular_fit_centre_y, 2) + crab.bg_Euclidean_AB_dis
t +      crab_area + (1 | sessionID)
## Fixed coefficients
##                                               coef    exp(coef)
## screenScale                            3.422238e-01 1.408075e+00
## playedBeforey                          4.583236e-01 1.581421e+00
## poly(crab_circular_fit_centre_x, 2)1 -1.368549e+01 1.138847e-06
## poly(crab_circular_fit_centre_x, 2)2 -3.881464e+01 1.390005e-17
## poly(crab_circular_fit_centre_y, 2)1 -1.054609e+01 2.629619e-05
## poly(crab_circular_fit_centre_y, 2)2 -2.987433e+01 1.061072e-13
## crab.bg_Euclidean_AB_dist              6.446571e-02 1.066589e+00
## crab_area                              1.944167e-05 1.000019e+00
##                                          se(coef)      z     p
## screenScale                           5.433208e-02   6.30 3e-10
## playedBeforey                         3.080031e-02  14.88 0e+00
## poly(crab_circular_fit_centre_x, 2)1 1.062848e+00 -12.88 0e+00
## poly(crab_circular_fit_centre_x, 2)2 1.085514e+00 -35.76 0e+00
## poly(crab_circular_fit_centre_y, 2)1 1.067560e+00  -9.88 0e+00
## poly(crab_circular_fit_centre_y, 2)2 1.095265e+00 -27.28 0e+00
## crab.bg_Euclidean_AB_dist             1.680718e-03  38.36 0e+00
## crab_area                             4.225988e-07  46.01 0e+00
##
## Random effects
##  Group     Variable  Std Dev   Variance
##  sessionID Intercept 0.5985476 0.3582593
```

```
# Best Colour = crab.bg_Euclidean_AB_dist


# Best Disruption = L_GabRat_sig3.0
# Best Pattern = crab.surr_DoGdiff
# Best Luminance = surr_L_diff
# Best Colour = crab.bg_Euclidean_AB_dist

# The overall best predictor is GabRat

# ------------------- Camouflage interaction with encounters
--------------------------------

# Disruption (L_GabRat_sig3.0)
# e1 <- coxme(Surv(cTime, hit) ~ screenScale + playedBefore + slide + crab_area +
poly(crab_circular_fit_centre_x,2) + poly(crab_circular_fit_centre_y,2) + L_GabRat
_sig3.0 * encounters + (1|sessionID) + (1|bgID), data)
# summary(e1) # z=  1.72 p=8.5e-02 NS - far too slow to model, adding bg as random
effect doens't seem useful

e1.1 <- coxme(Surv(cTime, hit) ~ screenScale + playedBefore + slide + crab_area +
poly(crab_circular_fit_centre_x,2) + poly(crab_circular_fit_centre_y,2) + L_GabRat
_sig3.0 * encounters + (1|sessionID), data)
summary(e1.1) # z = 1.64 p = 1.0e-01
```

```
## Cox mixed-effects model fit by maximum likelihood
##   Data: data
##   events, n = 40354, 42023
##   Iterations= 13 73
##                     NULL Integrated  Fitted
## Log-likelihood -394639.4  -385087.7 -382238
##
##                       Chisq      df p      AIC      BIC
## Integrated loglik 19103.41   12.00 0 19079.41 18976.15
##  Penalized loglik 24802.82 1589.89 0 21623.04  7941.31
##
## Model:  Surv(cTime, hit) ~ screenScale + playedBefore + slide + crab_area +
poly(crab_circular_fit_centre_x, 2) + poly(crab_circular_fit_centre_y,      2) + L
_GabRat_sig3.0 * encounters + (1 | sessionID)
## Fixed coefficients
##                                              coef    exp(coef)
## screenScale                          2.342069e-01 1.263906e+00
## playedBeforey                        4.600136e-01 1.584096e+00
## slide                                5.976739e-03 1.005995e+00
## crab_area                            1.940275e-05 1.000019e+00
## poly(crab_circular_fit_centre_x, 2)1 -1.391010e+01 9.097494e-07
## poly(crab_circular_fit_centre_x, 2)2 -4.240199e+01 3.846371e-19
## poly(crab_circular_fit_centre_y, 2)1 -1.084547e+01 1.949264e-05
## poly(crab_circular_fit_centre_y, 2)2 -3.296670e+01 4.816620e-15
## L_GabRat_sig3.0                      -7.409584e+00 6.054228e-04
## encounters                           5.268142e-02 1.054094e+00
## L_GabRat_sig3.0:encounters           5.076510e-02 1.052076e+00
##                                         se(coef)      z       p
## screenScale                          5.624755e-02   4.16 3.1e-05
## playedBeforey                        3.244990e-02  14.18 0.0e+00
## slide                                7.741890e-04   7.72 1.2e-14
## crab_area                            4.258458e-07  45.56 0.0e+00
## poly(crab_circular_fit_centre_x, 2)1 1.063497e+00 -13.08 0.0e+00
## poly(crab_circular_fit_centre_x, 2)2 1.089535e+00 -38.92 0.0e+00
## poly(crab_circular_fit_centre_y, 2)1 1.067857e+00 -10.16 0.0e+00
## poly(crab_circular_fit_centre_y, 2)2 1.100127e+00 -29.97 0.0e+00
## L_GabRat_sig3.0                      1.770909e-01 -41.84 0.0e+00
## encounters                           7.928161e-03   6.64 3.0e-11
## L_GabRat_sig3.0:encounters           3.097246e-02   1.64 1.0e-01
##
## Random effects
##  Group     Variable  Std Dev   Variance
##  sessionID Intercept 0.6352729 0.4035716
```

```
e1.2 <- coxme(Surv(cTime, hit) ~ screenScale + playedBefore + slide + crab_area +
poly(crab_circular_fit_centre_x,2) + poly(crab_circular_fit_centre_y,2) + A_GabRat
_sig4.0 * encounters + (1|sessionID), data)
summary(e1.2) # t = -3.06 p = 2.2e-03
```

```
## Cox mixed-effects model fit by maximum likelihood
##   Data: data
##   events, n = 40354, 42023
##   Iterations= 13 73
##                     NULL Integrated    Fitted
## Log-likelihood -394639.4  -386167.8 -383378.4
##
##                      Chisq      df p      AIC      BIC
## Integrated loglik 16943.10   12.00 0 16919.10 16815.83
##  Penalized loglik 22522.02 1580.63 0 19360.75  5758.69
##
## Model:  Surv(cTime, hit) ~ screenScale + playedBefore + slide + crab_area +
poly(crab_circular_fit_centre_x, 2) + poly(crab_circular_fit_centre_y,      2) + A
_GabRat_sig4.0 * encounters + (1 | sessionID)
## Fixed coefficients
##                                               coef    exp(coef)
## screenScale                            1.912739e-01 1.210791e+00
## playedBeforey                          4.419867e-01 1.555795e+00
## slide                                  5.314549e-03 1.005329e+00
## crab_area                              2.014650e-05 1.000020e+00
## poly(crab_circular_fit_centre_x, 2)1 -1.126590e+01 1.280206e-05
## poly(crab_circular_fit_centre_x, 2)2 -4.038292e+01 2.896822e-18
## poly(crab_circular_fit_centre_y, 2)1 -9.890506e+00 5.065332e-05
## poly(crab_circular_fit_centre_y, 2)2 -3.095360e+01 3.605954e-14
## A_GabRat_sig4.0                       -3.020475e+00 4.877803e-02
## encounters                             8.602056e-02 1.089829e+00
## A_GabRat_sig4.0:encounters            -7.027170e-02 9.321405e-01
##                                          se(coef)      z       p
## screenScale                           5.603200e-02   3.41 6.4e-04
## playedBeforey                         3.166390e-02  13.96 0.0e+00
## slide                                 7.724452e-04   6.88 6.0e-12
## crab_area                             4.336159e-07  46.46 0.0e+00
## poly(crab_circular_fit_centre_x, 2)1 1.064867e+00 -10.58 0.0e+00
## poly(crab_circular_fit_centre_x, 2)2 1.088162e+00 -37.11 0.0e+00
## poly(crab_circular_fit_centre_y, 2)1 1.067272e+00  -9.27 0.0e+00
## poly(crab_circular_fit_centre_y, 2)2 1.097069e+00 -28.21 0.0e+00
## A_GabRat_sig4.0                       1.301893e-01 -23.20 0.0e+00
## encounters                            6.985680e-03  12.31 0.0e+00
## A_GabRat_sig4.0:encounters            2.296689e-02  -3.06 2.2e-03
##
## Random effects
##  Group     Variable  Std Dev   Variance
##  sessionID Intercept 0.6169641 0.3806447
```

```
# Pattern (crab_meanPower)
e2 <- coxme(Surv(cTime, hit) ~ screenScale + playedBefore + slide + poly(crab_circ
ular_fit_centre_x,2) + poly(crab_circular_fit_centre_y,2) + crab.surr_DoGdiff * en
counters + crab_area + (1|sessionID), data)
summary(e2) # z= 0.82 p=4.1e-01 NS interaction
```

```
## Cox mixed-effects model fit by maximum likelihood
##   Data: data
##   events, n = 40354, 42023
##   Iterations= 13 73
##                     NULL Integrated    Fitted
## Log-likelihood -394639.4  -386789.4 -384005.4
##
##                      Chisq      df p      AIC      BIC
## Integrated loglik 15699.94   12.00 0 15675.94 15572.68
##  Penalized loglik 21267.86 1579.72 0 18108.42  4514.25
##
## Model:  Surv(cTime, hit) ~ screenScale + playedBefore + slide + poly(crab_circu
lar_fit_centre_x,      2) + poly(crab_circular_fit_centre_y, 2) + crab.surr_DoGdif
f *      encounters + crab_area + (1 | sessionID)
## Fixed coefficients
##                                              coef    exp(coef)
## screenScale                          1.981635e-01 1.219162e+00
## playedBeforey                        4.285304e-01 1.535000e+00
## slide                                4.463895e-03 1.004474e+00
## poly(crab_circular_fit_centre_x, 2)1 -1.349628e+01 1.376063e-06
## poly(crab_circular_fit_centre_x, 2)2 -3.871109e+01 1.541659e-17
## poly(crab_circular_fit_centre_y, 2)1 -1.052714e+01 2.679907e-05
## poly(crab_circular_fit_centre_y, 2)2 -3.033499e+01 6.693925e-14
## crab.surr_DoGdiff                    1.541403e-02 1.015533e+00
## encounters                           6.213153e-02 1.064102e+00
## crab_area                            2.499538e-05 1.000025e+00
## crab.surr_DoGdiff:encounters         4.184385e-04 1.000419e+00
##                                         se(coef)      z       p
## screenScale                         5.610567e-02   3.53 4.1e-04
## playedBeforey                       3.159354e-02  13.56 0.0e+00
## slide                               7.745585e-04   5.76 8.3e-09
## poly(crab_circular_fit_centre_x, 2)1 1.062736e+00 -12.70 0.0e+00
## poly(crab_circular_fit_centre_x, 2)2 1.085979e+00 -35.65 0.0e+00
## poly(crab_circular_fit_centre_y, 2)1 1.066996e+00  -9.87 0.0e+00
## poly(crab_circular_fit_centre_y, 2)2 1.097165e+00 -27.65 0.0e+00
## crab.surr_DoGdiff                   2.890379e-03   5.33 9.7e-08
## encounters                          3.283817e-03  18.92 0.0e+00
## crab_area                           4.151038e-07  60.21 0.0e+00
## crab.surr_DoGdiff:encounters        5.077251e-04   0.82 4.1e-01
##
## Random effects
##  Group     Variable  Std Dev   Variance
##  sessionID Intercept 0.6152046 0.3784768
```

```
# Luminance (surr_L_diff)
e3 <- coxme(Surv(cTime, hit) ~ screenScale + playedBefore + slide + poly(crab_circ
ular_fit_centre_x,2) + poly(crab_circular_fit_centre_y,2) + surr_L_diff * encounte
rs + crab_area + (1|sessionID), data)
summary(e3) # z= 1.12 p=2.6e-01 NS interaction
```

```
## Cox mixed-effects model fit by maximum likelihood
##   Data: data
##   events, n = 40354, 42023
##   Iterations= 13 73
##                     NULL Integrated    Fitted
## Log-likelihood -394639.4  -385178.6 -382335.3
##
##                       Chisq      df p      AIC      BIC
## Integrated loglik 18921.46   12.00 0 18897.46 18794.19
##  Penalized loglik 24608.20 1588.92 0 21430.35  7756.95
##
## Model:  Surv(cTime, hit) ~ screenScale + playedBefore + slide + poly(crab_circu
lar_fit_centre_x,      2) + poly(crab_circular_fit_centre_y, 2) + surr_L_diff *
encounters + crab_area + (1 | sessionID)
## Fixed coefficients
##                                               coef    exp(coef)
## screenScale                           2.057301e-01 1.228422e+00
## playedBeforey                         4.621349e-01 1.587459e+00
## slide                                 6.030685e-03 1.006049e+00
## poly(crab_circular_fit_centre_x, 2)1 -1.394125e+01 8.818430e-07
## poly(crab_circular_fit_centre_x, 2)2 -4.151003e+01 9.384711e-19
## poly(crab_circular_fit_centre_y, 2)1 -1.150550e+01 1.007449e-05
## poly(crab_circular_fit_centre_y, 2)2 -3.269961e+01 6.291321e-15
## surr_L_diff                           8.321897e-01 2.298346e+00
## encounters                            6.221684e-02 1.064193e+00
## crab_area                             2.327387e-05 1.000023e+00
## surr_L_diff:encounters                4.249073e-03 1.004258e+00
##                                          se(coef)      z       p
## screenScale                          5.660491e-02   3.63 2.8e-04
## playedBeforey                        3.233529e-02  14.29 0.0e+00
## slide                                7.750917e-04   7.78 7.2e-15
## poly(crab_circular_fit_centre_x, 2)1 1.063550e+00 -13.11 0.0e+00
## poly(crab_circular_fit_centre_x, 2)2 1.088854e+00 -38.12 0.0e+00
## poly(crab_circular_fit_centre_y, 2)1 1.067972e+00 -10.77 0.0e+00
## poly(crab_circular_fit_centre_y, 2)2 1.098393e+00 -29.77 0.0e+00
## surr_L_diff                          2.144879e-02  38.80 0.0e+00
## encounters                           3.950231e-03  15.75 0.0e+00
## crab_area                            4.176626e-07  55.72 0.0e+00
## surr_L_diff:encounters               3.781335e-03   1.12 2.6e-01
##
## Random effects
##  Group     Variable  Std Dev   Variance
##  sessionID Intercept 0.6333066 0.4010773
```

```
# Colour (crab.bg_Euclidean_AB_dist)
e4 <- coxme(Surv(cTime, hit) ~ screenScale + playedBefore + slide + poly(crab_circ
ular_fit_centre_x,2) + poly(crab_circular_fit_centre_y,2) + crab.bg_Euclidean_AB_d
ist * encounters + crab_area + (1|sessionID), data)
summary(e4) # z= 3.34 p=8.4e-04 Significant interaction
```

```
## Cox mixed-effects model fit by maximum likelihood
##   Data: data
##   events, n = 40354, 42023
##   Iterations= 22 140
##                     NULL Integrated    Fitted
## Log-likelihood -394639.4  -386073.9 -383285.1
##
##                       Chisq      df p      AIC      BIC
## Integrated loglik 17130.84   12.00 0 17106.84 17003.57
##  Penalized loglik 22708.63 1582.22 0 19544.19  5928.51
##
## Model:  Surv(cTime, hit) ~ screenScale + playedBefore + slide + poly(crab_circu
lar_fit_centre_x,      2) + poly(crab_circular_fit_centre_y, 2) + crab.bg_Euclidea
n_AB_dist *      encounters + crab_area + (1 | sessionID)
## Fixed coefficients
##                                              coef    exp(coef)
## screenScale                          1.844307e-01 1.202534e+00
## playedBeforey                        4.484547e-01 1.565890e+00
## slide                                5.904070e-03 1.005922e+00
## poly(crab_circular_fit_centre_x, 2)1 -1.372288e+01 1.097058e-06
## poly(crab_circular_fit_centre_x, 2)2 -4.001494e+01 4.185368e-18
## poly(crab_circular_fit_centre_y, 2)1 -1.076242e+01 2.118080e-05
## poly(crab_circular_fit_centre_y, 2)2 -3.100541e+01 3.423891e-14
## crab.bg_Euclidean_AB_dist             6.073682e-02 1.062619e+00
## encounters                            5.568480e-02 1.057264e+00
## crab_area                             2.034602e-05 1.000020e+00
## crab.bg_Euclidean_AB_dist:encounters  1.415141e-03 1.001416e+00
##                                          se(coef)      z       p
## screenScale                          5.623345e-02   3.28 1.0e-03
## playedBeforey                        3.179409e-02  14.10 0.0e+00
## slide                                7.729760e-04   7.64 2.2e-14
## poly(crab_circular_fit_centre_x, 2)1 1.063346e+00 -12.91 0.0e+00
## poly(crab_circular_fit_centre_x, 2)2 1.087283e+00 -36.80 0.0e+00
## poly(crab_circular_fit_centre_y, 2)1 1.067918e+00 -10.08 0.0e+00
## poly(crab_circular_fit_centre_y, 2)2 1.097388e+00 -28.25 0.0e+00
## crab.bg_Euclidean_AB_dist            2.388459e-03  25.43 0.0e+00
## encounters                           2.984612e-03  18.66 0.0e+00
## crab_area                            4.305070e-07  47.26 0.0e+00
## crab.bg_Euclidean_AB_dist:encounters 4.237485e-04   3.34 8.4e-04
##
## Random effects
##  Group     Variable  Std Dev   Variance
##  sessionID Intercept 0.6200436 0.3844541
```

```
#
# # checking residuals using GLMs
# m <- glm(log(cTime) ~ screenScale + playedBefore + poly(crab_circular_fit_centre
_x,2) + poly(crab_circular_fit_centre_y,2) + crab.bg_Euclidean_AB_dist * log(encou
nters) + crab_area, data=data)
# plot(m)
# m1 <- glm(log(cTime) ~ screenScale + playedBefore + poly(crab_circular_fit_centr
e_x,2) + poly(crab_circular_fit_centre_y,2) + crab.bg_Euclidean_AB_dist * encounte
rs  + crab_area, data=data)
# plot(m1)
# m2 <- glm(log(cTime) ~ screenScale + playedBefore + poly(crab_circular_fit_centr
e_x,2) + poly(crab_circular_fit_centre_y,2) + crab.bg_Euclidean_AB_dist + crab_are
a, data=data)
# plot(m2)
# # all look good
#
# m <- glm(log(cTime) ~ log(encounters), data=data)
# plot(m) # looks like encounters works ok
# m <- glm(log(cTime) ~ encounters, data=data)
# plot(m) # looks like encounters works ok without log

# Everything except luminance has significant interaction with previous number of
encounters

# -----------------------Plotting------------------------------


data$drpQ <- quantcut(data$A_GabRat_sig4.0, q=2)

# Disrupitve
for(i in 1:length(data$crabSeq)){
    if(data$encounters[i] == 1){
        if(data$drpQ[i] == levels(data$drpQ)[1]){
            data$drpQe[i] <- "Novel|Low Disruption"
        } else data$drpQe[i] <- "Novel|High Disruption"
    }else if(data$drpQ[i] == levels(data$drpQ)[1]){
            data$drpQe[i] <- "Repeat|Low Disruption"
        } else data$drpQe[i] <- "Repeat|High Disruption"
}
data$drpQe <- factor(data$drpQe)

sPlot <- survfit(Surv(cTime, hit) ~ drpQe, data)
plot(sPlot, conf.int=TRUE, col=c("red","Dark Red", "blue", "Dark Blue"), lty=1:2,
xlab="Time (ms)", ylab="Survival")
legend(8000, 1, levels(data$drpQe), col=c("red","Dark Red", "blue", "Dark Blue"),
lty = 1:2)
```

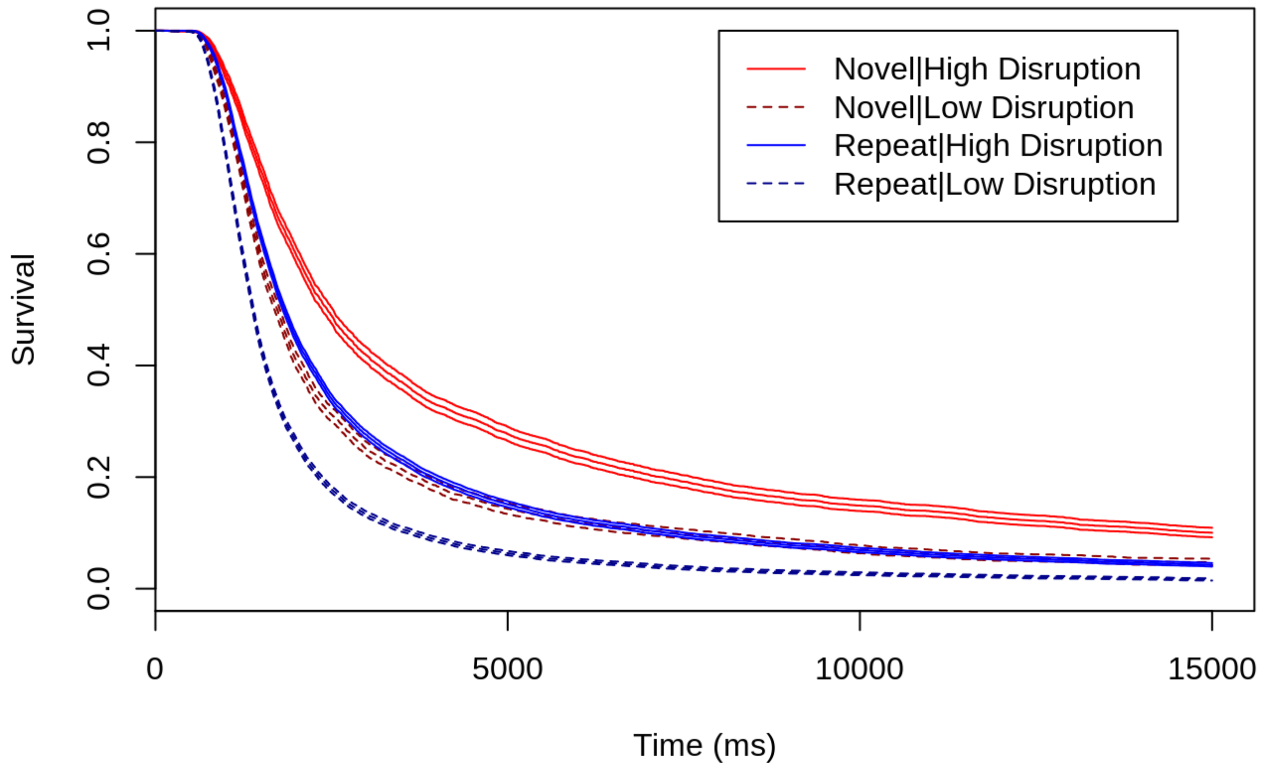


```
ggplot(data=data, aes(factor(encounters), cTime, color=drpQ)) +
  geom_boxplot() +
  geom_smooth(method=lm, se=TRUE, aes(group=drpQ)) +
  labs(y="Capture Time (ms)")+
  labs(x="Sequential Encounters")+
  scale_y_continuous(trans = "log", breaks =c(125,250,500,1000,2000,4000,8000,1600
0)) +
  theme(panel.grid.major = element_blank(), panel.grid.minor = element_blank(), pa
nel.background = element_blank(), panel.border = element_rect(colour = "black", fi
ll=NA) , legend.position=c(0.8,0.2))
```

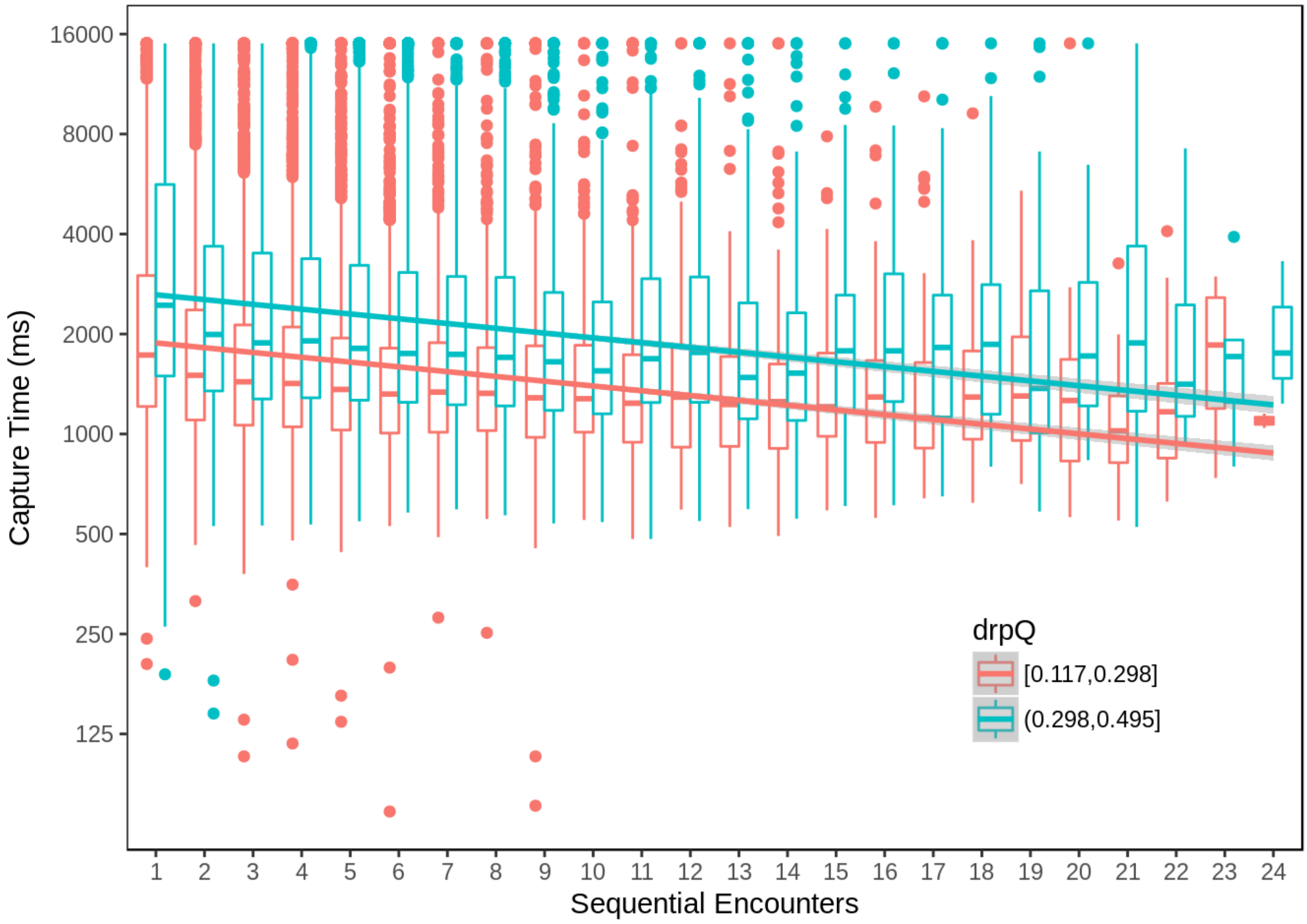


```
# Pattern
data$patQ <- quantcut(data$crab.surr_DoGdiff, q=2)
data$patQe <- as.character(data$patQ)

for(i in 1:length(data$crabSeq)){
    if(data$encounters[i] == 1){
        if(data$patQ[i] == levels(data$patQ)[1]){
            data$patQe[i] <- "Novel|Good Pattern Match"
        } else data$patQe[i] <- "Novel|Poor Pattern Match"
    }else if(data$patQ[i] == levels(data$patQ)[1]){
            data$patQe[i] <- "Repeat|Good Pattern Match"
        } else data$patQe[i] <- "Repeat|Poor Pattern Match"
}

data$patQe <- factor(data$patQe)

sPlot <- survfit(Surv(cTime, hit) ~ patQe , data)
plot(sPlot, conf.int=TRUE, col=c("red","Dark Red", "blue", "Dark Blue"), lty=1:2,
xlab="Time (ms)", ylab="Survival")
legend(8000, 1, levels(data$patQe), col=c("red","Dark Red", "blue", "Dark Blue"),
lty = 1:2)
```

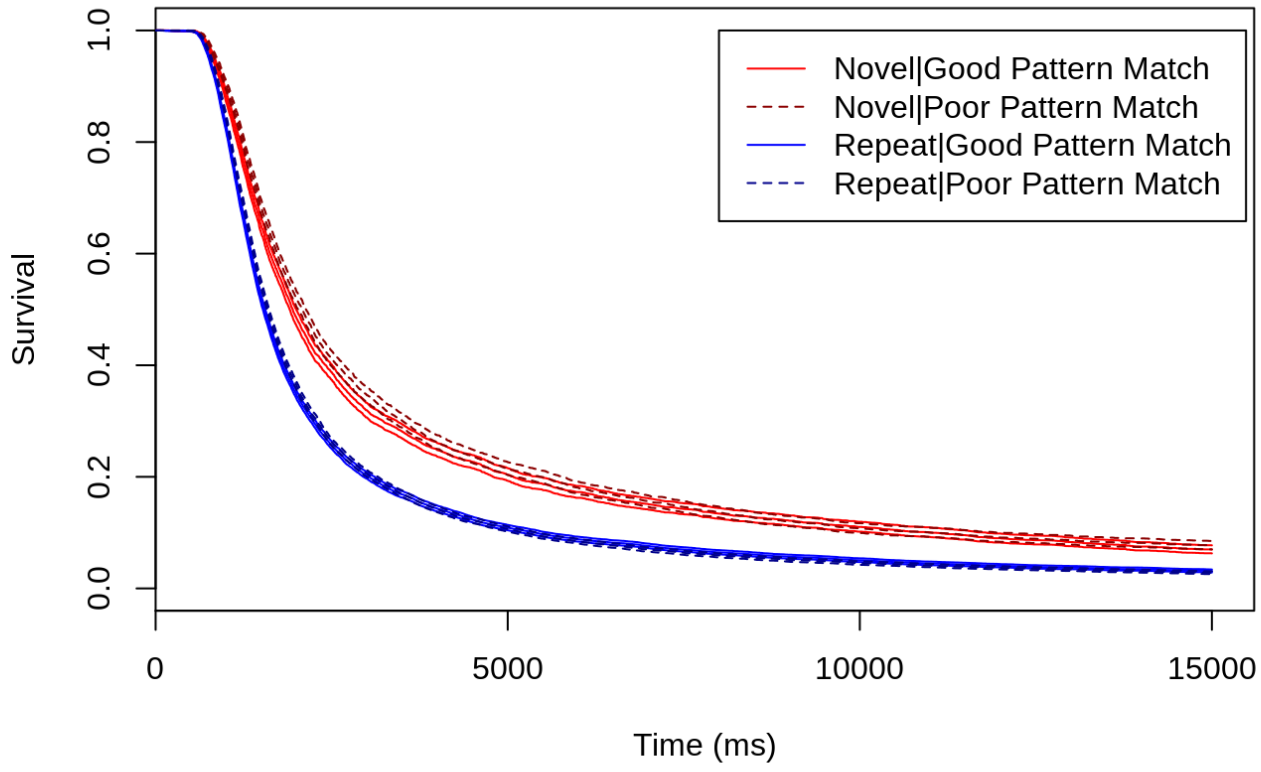


```
ggplot(data=data, aes(factor(encounters), cTime, color=patQ)) +
  geom_boxplot() +
  geom_smooth(method=lm, se=TRUE, aes(group=patQ)) +
  labs(y="Capture Time (s)")+
  labs(x="Sequential Encounters")+
  scale_y_continuous(trans = "log", breaks =c(125,250,500,1000,2000,4000,8000,1600
0)) +
  theme(panel.grid.major = element_blank(), panel.grid.minor = element_blank(), pa
nel.background = element_blank(), panel.border = element_rect(colour = "black", fi
ll=NA) , legend.position=c(0.8,0.2))
```

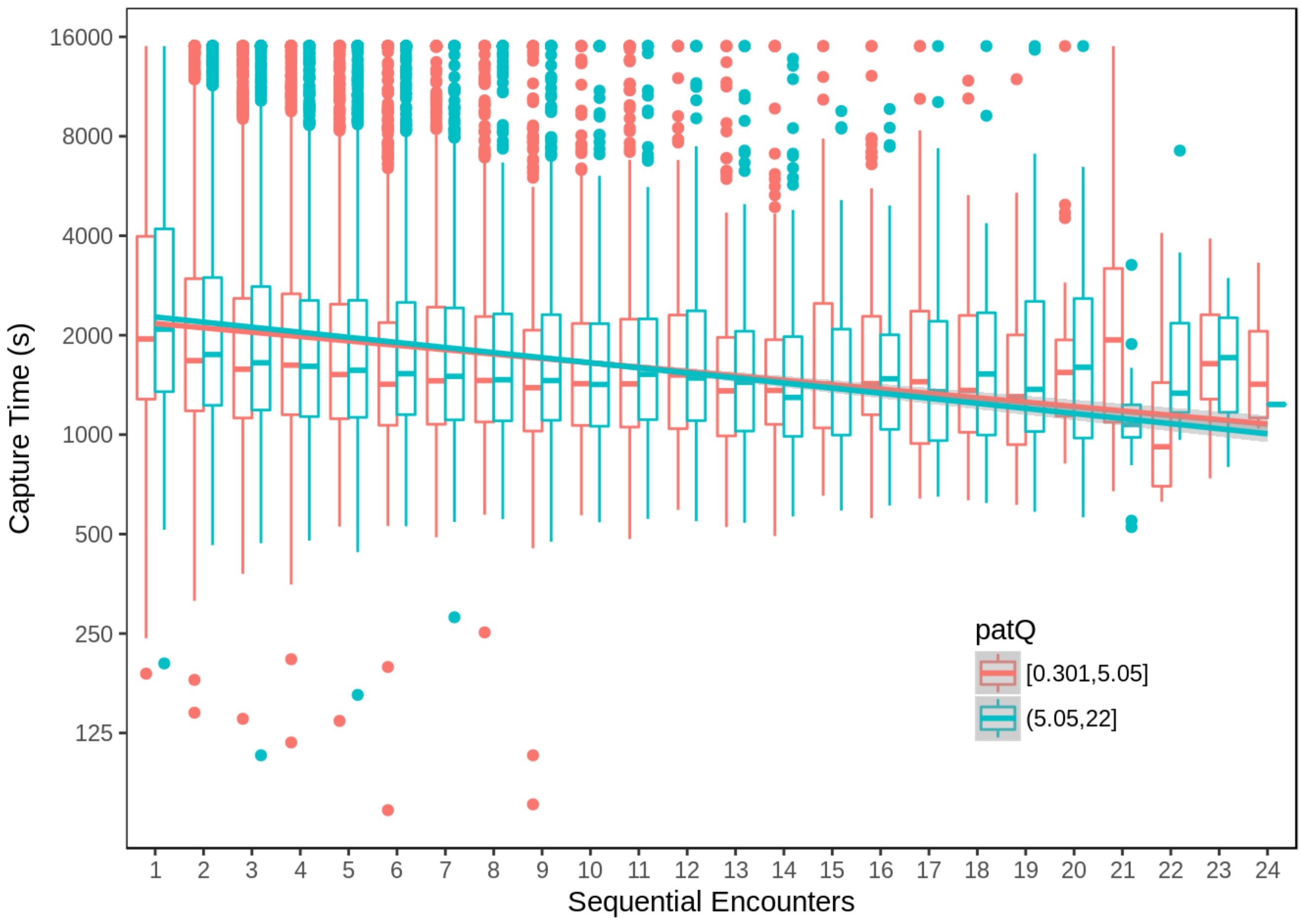


```
# Luminance
data$lumQ <- quantcut(data$surr_L_diff, q=2)

for(i in 1:length(data$crabSeq)){
    if(data$encounters[i] == 1){
        if(data$lumQ[i] == levels(data$lumQ)[1]){
            data$lumQe[i] <- "Novel|Good Luminance Match"
        } else data$lumQe[i] <- "Novel|Poor Luminance Match"
    }else if(data$lumQ[i] == levels(data$lumQ)[1]){
            data$lumQe[i] <- "Repeat|Good Luminance Match"
        } else data$lumQe[i] <- "Repeat|Poor Luminance Match"
}

data$lumQe <- factor(data$lumQe)

sPlot <- survfit(Surv(cTime, hit) ~ lumQe , data)
plot(sPlot, conf.int=TRUE, col=c("red","Dark Red", "blue", "Dark Blue"), lty=1:2,
xlab="Time (ms)", ylab="Survival")
legend(8000, 1, levels(data$lumQe), col=c("red","Dark Red", "blue", "Dark Blue"),
lty = 1:2)
```

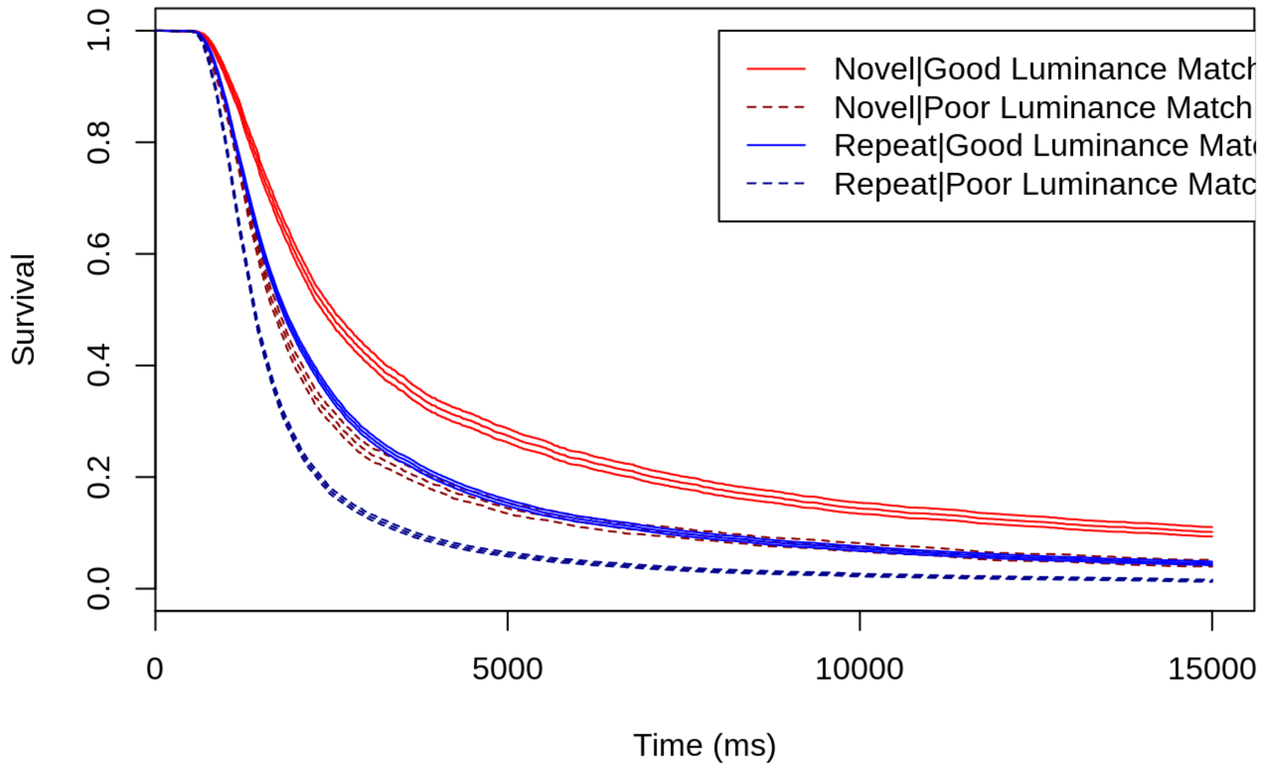


```
ggplot(data=data, aes(factor(encounters), cTime, color=lumQ)) +
  geom_boxplot() +
  geom_smooth(method=lm, se=TRUE, aes(group=lumQ)) +
  labs(y="Capture Time (s)")+
  labs(x="Sequential Encounters")+
  scale_y_continuous(trans = "log", breaks =c(125,250,500,1000,2000,4000,8000,1600
0)) +
  theme(panel.grid.major = element_blank(), panel.grid.minor = element_blank(), pa
nel.background = element_blank(), panel.border = element_rect(colour = "black", fi
ll=NA) , legend.position=c(0.8,0.2))
```

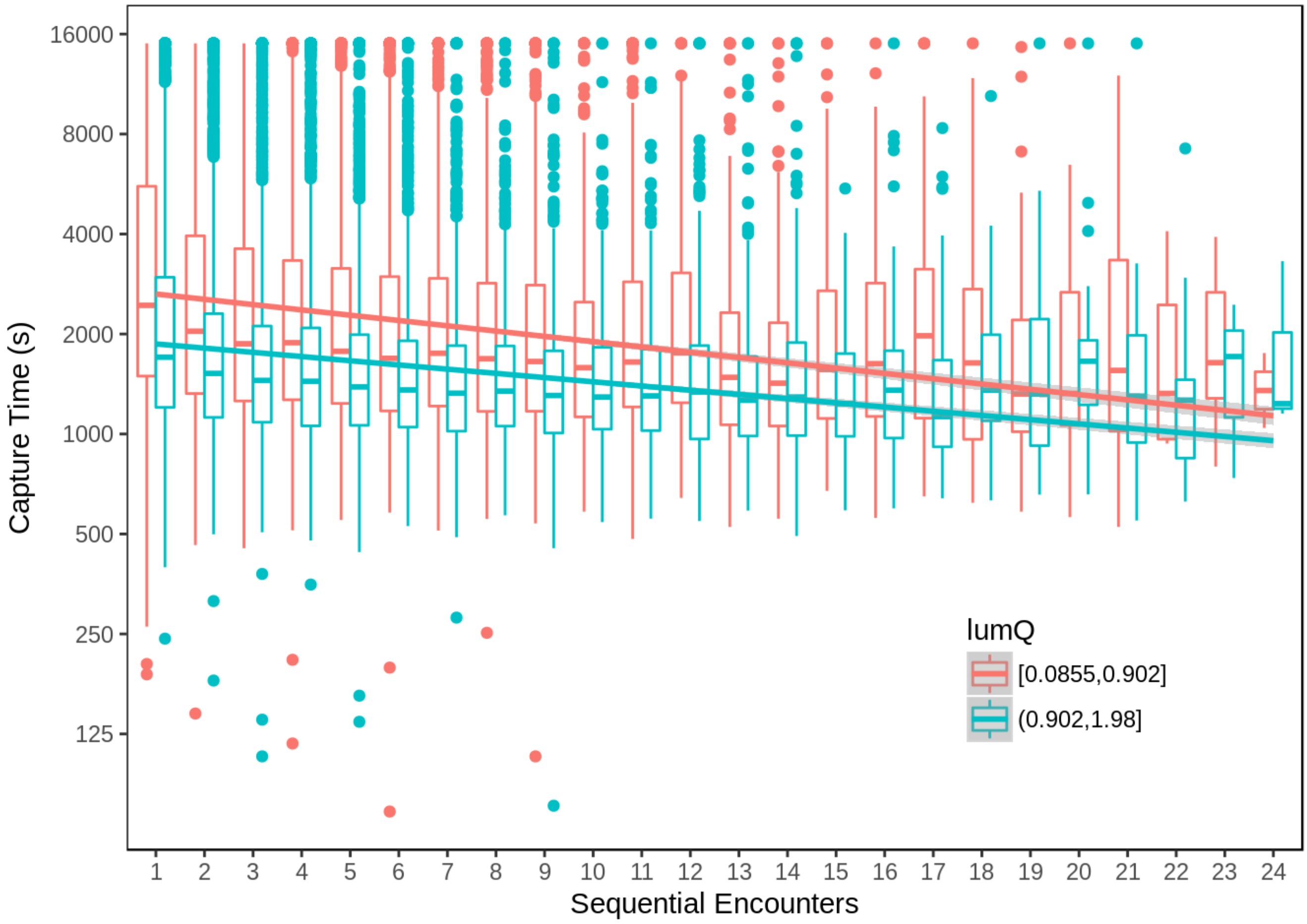


```
# Colour
data$colQ <- quantcut(data$crab.bg_Euclidean_AB_dist, q=2)

for(i in 1:length(data$crabSeq)){
    if(data$encounters[i] == 1){
        if(data$colQ[i] == levels(data$colQ)[1]){
            data$colQe[i] <- "Novel|Good Colour Match"
        } else data$colQe[i] <- "Novel|Poor Colour Match"
    }else if(data$colQ[i] == levels(data$colQ)[1]){
            data$colQe[i] <- "Repeat|Good Colour Match"
        } else data$colQe[i] <- "Repeat|Poor Colour Match"
}

data$colQe <- factor(data$colQe)

sPlot <- survfit(Surv(cTime, hit) ~ colQe , data)
plot(sPlot, conf.int=TRUE, col=c("red","Dark Red", "blue", "Dark Blue"), lty=1:2,
xlab="Time (ms)", ylab="Survival")
legend(8000, 1, levels(data$colQe), col=c("red","Dark Red", "blue", "Dark Blue"),
lty = 1:2)
```

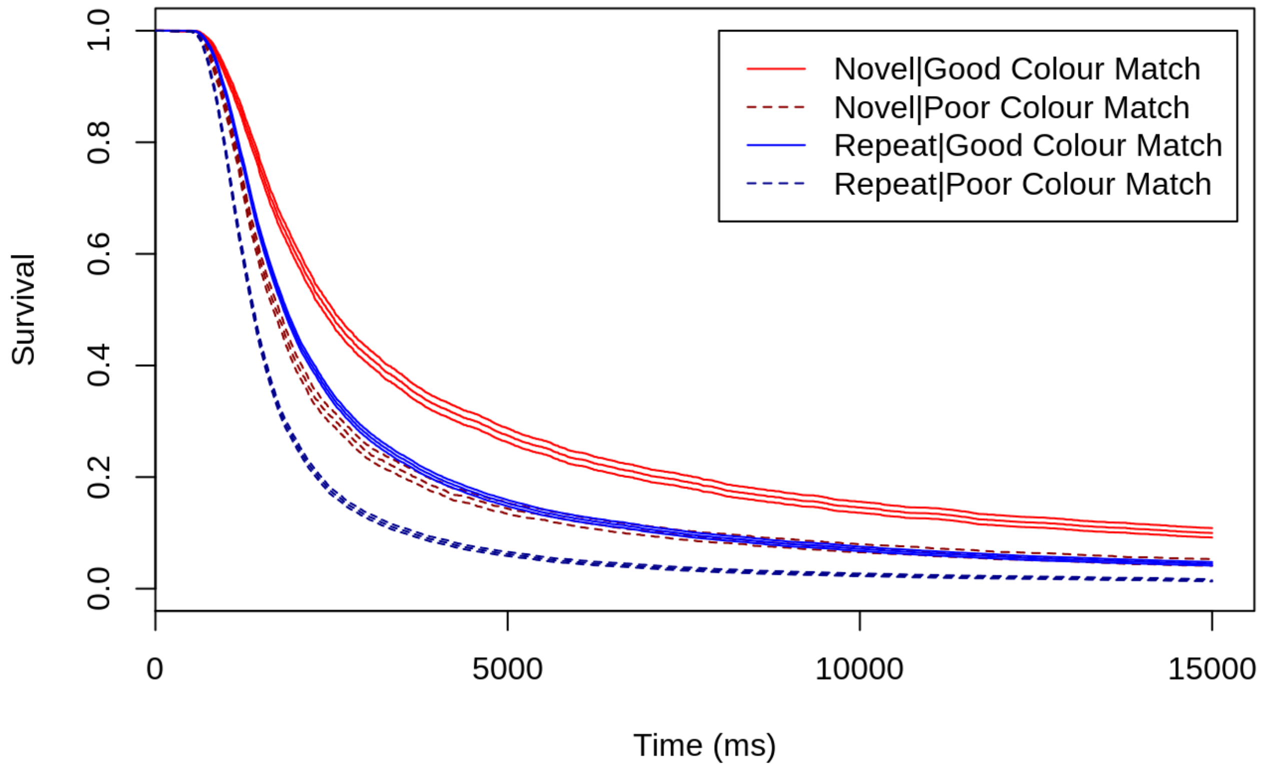


```
ggplot(data=data, aes(factor(encounters), cTime, color=colQ)) +
  geom_boxplot() +
  geom_smooth(method=lm, se=TRUE, aes(group=colQ)) +
  labs(y="Capture Time (s)")+
  labs(x="Sequential Encounters")+
  scale_y_continuous(trans = "log", breaks =c(125,250,500,1000,2000,4000,8000,1600
0)) +
  theme(panel.grid.major = element_blank(), panel.grid.minor = element_blank(), pa
nel.background = element_blank(), panel.border = element_rect(colour = "black", fi
ll=NA) , legend.position=c(0.8,0.2))
```

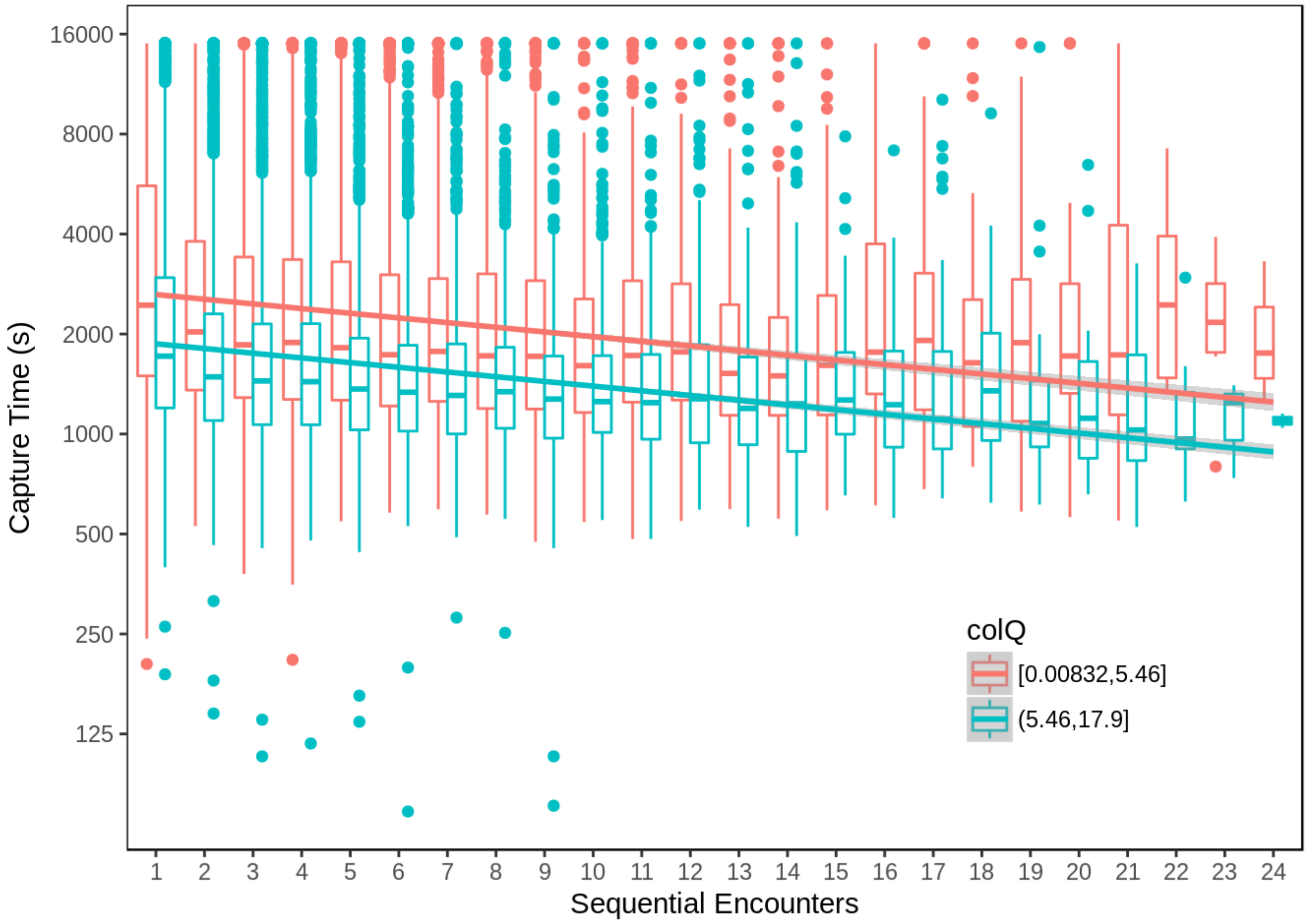


```
# --------------------------Switch event single-slide difference values
------------------------------

#  create morph switching categories for later
data$morphCode <- "NA"

for(i in 2:(length(data$crabSeq))){
  data$morphCode[i] <- paste(data$crabMorph[i-1], "_", data$crabMorph[i])
}

data$morphCode <- factor(data$morphCode)
levels(data$morphCode)
```

```
##  [1] "black _ black"          "black _ disruptive"
##  [3] "black _ green"          "black _ mottled"
##  [5] "black _ pale"           "black _ spotted"
##  [7] "disruptive _ black"     "disruptive _ disruptive"
##  [9] "disruptive _ green"     "disruptive _ mottled"
## [11] "disruptive _ pale"      "disruptive _ spotted"
## [13] "green _ black"          "green _ disruptive"
## [15] "green _ green"          "green _ mottled"
## [17] "green _ pale"           "green _ spotted"
## [19] "mottled _ black"        "mottled _ disruptive"
## [21] "mottled _ green"        "mottled _ mottled"
## [23] "mottled _ pale"         "mottled _ spotted"
## [25] "NA"                     "pale _ black"
## [27] "pale _ disruptive"      "pale _ green"
## [29] "pale _ mottled"         "pale _ pale"
## [31] "pale _ spotted"         "spotted _ black"
## [33] "spotted _ disruptive"   "spotted _ green"
## [35] "spotted _ mottled"      "spotted _ pale"
## [37] "spotted _ spotted"
```

```r
diffData <- data[,c("crabSeq", "slide", "cTime", "hit", "playedBefore","encounter
s", "sessionID", "crabID", "morphSwitch", "morphCode", "bgID", "crab_circular_fit_
centre_x", "crab_circular_fit_centre_y", "crab_circular_fit_diameter", "crab_are
a", "screenScale", "crab_L_mean", "crab_L_sd", "crab_A_mean", "crab_B_mean", "L_Ga
bRat_sig3.0", "A_GabRat_sig4.0", "crab_meanPower", "surr_L_diff", "crab.bg_Euclide
an_AB_dist", "crab.surr_DoGdiff")]

diffData$pX <- 0
diffData$pY <- 0
diffData$pArea <- 0
diffData$timeDiff <- 0
diffData$drpLDiff <- 0
diffData$drpADiff <- 0
diffData$colDiff <- 0
diffData$lumDiff <- 0
diffData$patDiff <- 0
diffData$change <- 0
diffData$novelCrab <- 0


for(i in 2:(length(diffData$crabSeq))){
  if(diffData$sessionID[i] == diffData$sessionID[i-1]){
    diffData$timeDiff[i] <- log(diffData$cTime[i]) - log(diffData$cTime[i-1])
    diffData$drpLDiff[i] <- diffData$L_GabRat_sig3.0[i] - diffData$L_GabRat_sig3.0
[i-1]
    diffData$drpADiff[i] <- diffData$A_GabRat_sig4.0[i] - diffData$A_GabRat_sig4.0
[i-1]
    diffData$colDiff[i] <- diffData$crab.bg_Euclidean_AB_dist[i] - diffData$crab.b
g_Euclidean_AB_dist[i-1]
    diffData$lumDiff[i] <- diffData$surr_L_diff[i] - diffData$surr_L_diff[i-1]
    diffData$patDiff[i] <- diffData$crab.surr_DoGdiff[i] - diffData$crab.surr_DoGd
iff[i-1]
    diffData$change[i] <- 1

    diffData$pX[i] <- diffData$crab_circular_fit_centre_x[i-1]
    diffData$pY[i] <- diffData$crab_circular_fit_centre_y[i-1]
    diffData$pArea[i] <- diffData$crab_area[i-1]

  }
}


diffData$novelCrab <- ifelse (diffData$encounters > 1, 0, 1)
diffData <- subset(diffData, change == 1)
diffData$novelCrab <- factor(diffData$novelCrab)


# str(diffData)

# --------------- Modelling capture time differences when switching between crabs
--------------------

# LUMINANCE DISRUPTION (& model format testing)
```

```
# df1 <- lmer(timeDiff ~ crab_area + pArea + screenScale + playedBefore + slide +
poly(crab_circular_fit_centre_x,2) + poly(crab_circular_fit_centre_y,2) + poly(pX,
2) + poly(pY,2) + drpLDiff*novelCrab + (1|sessionID), diffData)
# summary(df1) # screenScale can be dropped (makes sense given we're now lookign a
t time difference, so controlling for between-participant effects)
# mcp.fnc(df1) # look good - using log capture time results in much better residua
ls than using untransformed capture times.

# interestingly sessionID expalins no variance, suggesting a standard GLM could be
used instead. However, given the pseudoreplication
# between slides within a session this would probably be difficult to justify.


df1.1 <- lmer(timeDiff ~ crab_area + pArea + playedBefore + slide + poly(crab_circ
ular_fit_centre_x,2) + poly(crab_circular_fit_centre_y,2) + poly(pX,2) + poly(pY,
2) + drpLDiff*novelCrab + (1|sessionID), diffData)
summary(df1.1) # Significant interaciton between disruption differnece and novel c
rab t = 2.226  p = 0.02602 *
```

```
## Linear mixed model fit by REML ['lmerMod']
## Formula:
## timeDiff ~ crab_area + pArea + playedBefore + slide + poly(crab_circular_fit_ce
ntre_x,
##     2) + poly(crab_circular_fit_centre_y, 2) + poly(pX, 2) +
##     poly(pY, 2) + drpLDiff * novelCrab + (1 | sessionID)
##    Data: diffData
##
## REML criterion at convergence: 91081.7
##
## Scaled residuals:
##     Min      1Q  Median      3Q     Max
## -4.3947 -0.5089  0.0017  0.4989  4.4474
##
## Random effects:
##  Groups    Name        Variance Std.Dev.
##  sessionID (Intercept) 0.000    0.000
##  Residual              0.561    0.749
## Number of obs: 40272, groups:  sessionID, 1751
##
## Fixed effects:
##                                         Estimate Std. Error t value
## (Intercept)                           -1.472e-01  1.163e-02 -12.655
## crab_area                             -1.058e-05  4.410e-07 -23.995
## pArea                                  1.086e-05  4.405e-07  24.667
## playedBeforey                          2.052e-02  7.730e-03   2.655
## slide                                  5.214e-03  5.629e-04   9.262
## poly(crab_circular_fit_centre_x, 2)1  6.671e+00  7.496e-01   8.900
## poly(crab_circular_fit_centre_x, 2)2  2.310e+01  7.564e-01  30.545
## poly(crab_circular_fit_centre_y, 2)1  6.388e+00  7.509e-01   8.507
## poly(crab_circular_fit_centre_y, 2)2  1.774e+01  7.675e-01  23.107
## poly(pX, 2)1                          -7.016e+00  7.496e-01  -9.360
## poly(pX, 2)2                          -2.236e+01  7.565e-01 -29.562
## poly(pY, 2)1                          -6.562e+00  7.509e-01  -8.739
## poly(pY, 2)2                          -1.572e+01  7.673e-01 -20.493
## drpLDiff                               3.722e+00  7.173e-02  51.892
## novelCrab1                             2.258e-01  9.383e-03  24.070
## drpLDiff:novelCrab1                    3.320e-01  1.492e-01   2.226
```

```
##
## Correlation matrix not shown by default, as p = 16 > 12.
## Use print(x, correlation=TRUE)  or
##   vcov(x)     if you need it
```

```
## fit warnings:
## Some predictor variables are on very different scales: consider rescaling
```

```
mcp.fnc(df1.1) # resids look very nice, imply bounding issues aren't too bad, wiht
otherwise nice distribution
```

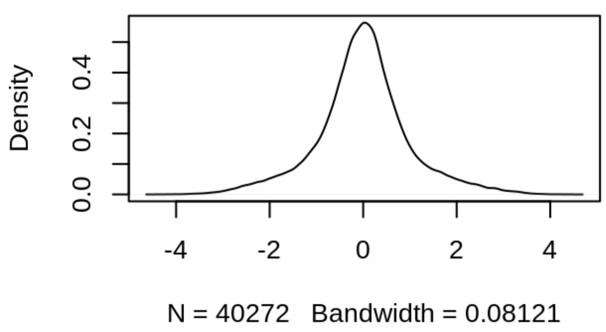

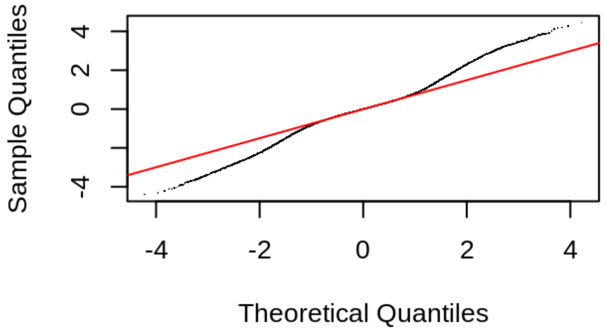

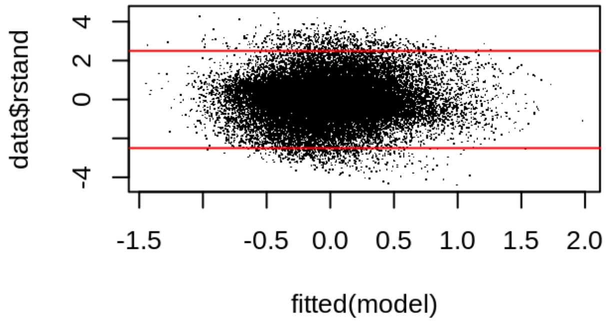


```
# anova(df1, df1.1) # no diff, keep screenscale out

# df1.2 <- lmer(timeDiff ~ crab_area + pArea + slide + poly(crab_circular_fit_cent
re_x,2) + poly(crab_circular_fit_centre_y,2) + poly(pX,2) + poly(pY,2) + drpLDiff*
novelCrab + (1|sessionID), diffData)
# summary(df1.2)
# anova(df1.1, df1.2) # model 1.1 is significantly better, so keep played before i
n the model

# df1.3 <- glm(timeDiff ~ crab_area + pArea + slide + poly(crab_circular_fit_centr
e_x,2) + poly(crab_circular_fit_centre_y,2) + poly(pX,2) + poly(pY,2) + drpLDiff*n
ovelCrab, data=diffData)
# summary(df1.3)
# Standard GLM makes no difference to result, still significant interaction

ggplot(diffData, aes(x=drpLDiff, y=timeDiff, color=novelCrab, shape=novelCrab)) +
geom_point(size = 0.5, alpha = 0.2) + # plot linearity
  scale_colour_hue(l=40) +
  geom_smooth(method=lm, se=TRUE)
```

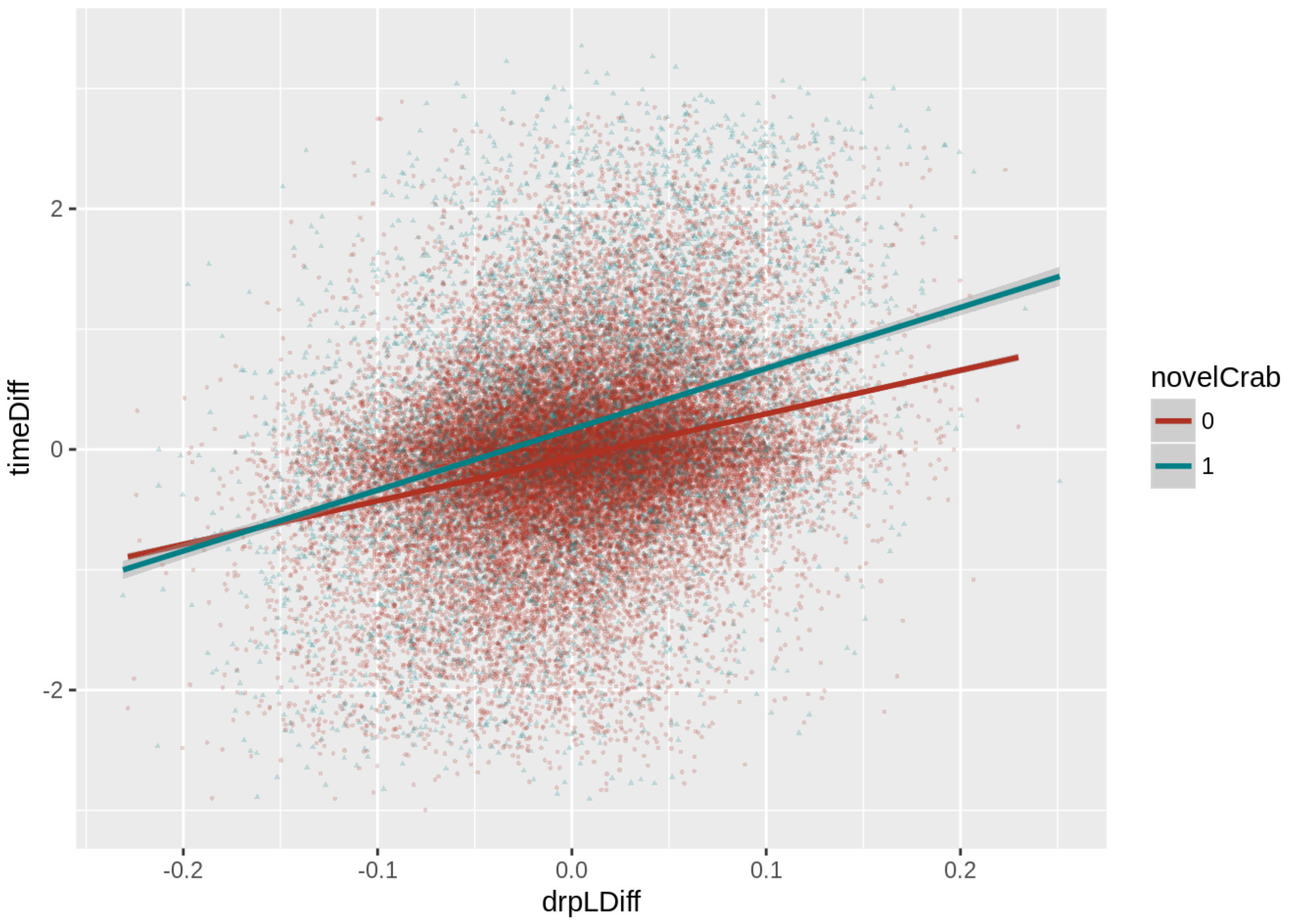


```
diffData$drpLDiffQ <- quantcut(diffData$drpLDiff, q=5)

ggplot(data=diffData, aes(drpLDiffQ, timeDiff, color=novelCrab)) +
  geom_boxplot() +
  geom_smooth(method=lm, se=TRUE, aes(group=novelCrab)) +
  labs(y="Capture time difference between sequential encounters (ms)")+
  labs(x="Disruption Difference between sequential encounters") +
  theme(panel.grid.major = element_blank(), panel.grid.minor = element_blank(), pa
nel.background = element_blank(), panel.border = element_rect(colour = "black", fi
ll=NA) , legend.position="top")
```

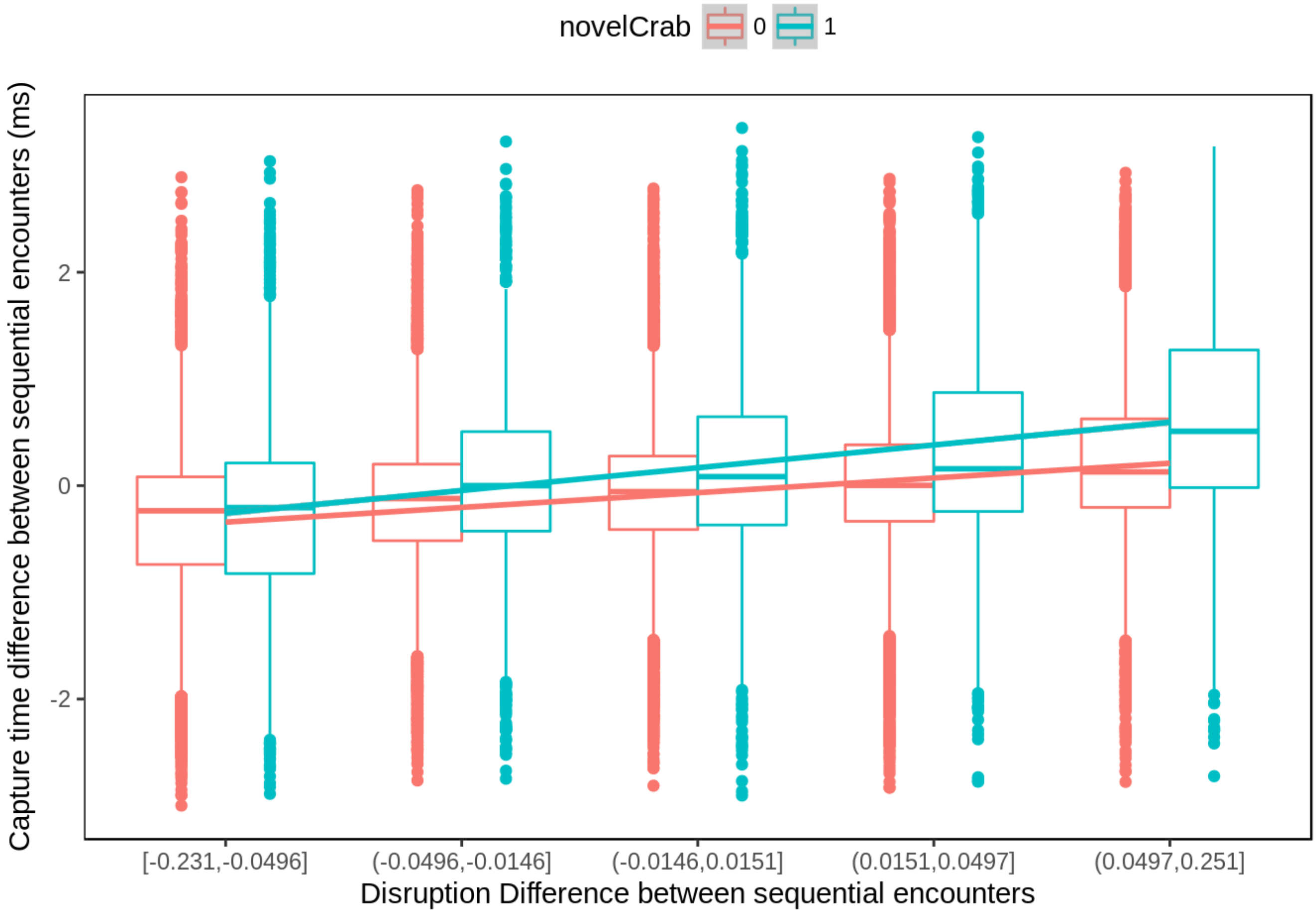


```
# effect size example

temp <- subset(diffData, diffData$drpLDiffQ == levels(diffData$drpLDiffQ)[1] )
temp <- subset(temp, temp$novelCrab == 1 )
exp(mean(temp$timeDiff))
```

```
## [1] 0.7541921
```

```
# Reduced sequential disruption, Novel crab
# 0.7541921

temp <- subset(diffData, diffData$drpLDiffQ == levels(diffData$drpLDiffQ)[1] )
temp <- subset(temp, temp$novelCrab == 0 )
exp(mean(temp$timeDiff))
```

```
## [1] 0.7056809
```

```
# Reduced sequential disruption, same crab
# 0.7056809

temp <- subset(diffData, diffData$drpLDiffQ == levels(diffData$drpLDiffQ)[5] )
temp <- subset(temp, temp$novelCrab == 1 )
exp(mean(temp$timeDiff))
```

```
## [1] 1.872917
```

```
# Increased sequential disruption, Novel crab
# 1.872917

temp <- subset(diffData, diffData$drpLDiffQ == levels(diffData$drpLDiffQ)[5] )
temp <- subset(temp, temp$novelCrab == 0 )
exp(mean(temp$timeDiff))
```

```
## [1] 1.278947
```

```
# Increased sequential disruption, same crab
# 1.278947

# When switching to a novel crab which has higher disruption than the previous cra
b (disruption difference in the 80th-100th centile)
# it increased capture times by 0.59397 seconds, as opposed to a 0.0485112 second
increase when swithcing to a novel
# crab with lower disruption (in the 0-20th centile).

mean(diffData$cTime)
```

```
## [1] 2813.243
```

```
# 2813.243

# ------------using 'encounters' instead of 'novelCrab'------------------
# Continuous value for number of prior encounters with the same crab, rather than
binomial

df1.4 <- lmer(timeDiff ~ crab_area + pArea + slide + poly(crab_circular_fit_centre
_x,2) + poly(crab_circular_fit_centre_y,2) + poly(pX,2) + poly(pY,2) +  slide + dr
pLDiff*encounters + (1|sessionID), diffData)
summary(df1.4) # highly significant interaction between time difference and disrup
tion difference with crab encounters
```

```
## Linear mixed model fit by REML ['lmerMod']
## Formula:
## timeDiff ~ crab_area + pArea + slide + poly(crab_circular_fit_centre_x,
##     2) + poly(crab_circular_fit_centre_y, 2) + poly(pX, 2) +
##     poly(pY, 2) + slide + drpLDiff * encounters + (1 | sessionID)
##    Data: diffData
##
## REML criterion at convergence: 91619
##
## Scaled residuals:
##     Min      1Q  Median      3Q     Max
## -4.1579 -0.5092 -0.0129  0.4950  4.4816
##
## Random effects:
##  Groups    Name        Variance Std.Dev.
##  sessionID (Intercept) 0.0000   0.000
##  Residual              0.5685   0.754
## Number of obs: 40272, groups:  sessionID, 1751
##
## Fixed effects:
##                                     Estimate Std. Error t value
## (Intercept)                       -8.162e-02  1.162e-02  -7.023
## crab_area                         -1.058e-05  4.361e-07 -24.259
## pArea                              1.085e-05  4.356e-07  24.915
## slide                              5.729e-03  5.798e-04   9.881
## poly(crab_circular_fit_centre_x, 2)1  6.665e+00  7.546e-01   8.832
## poly(crab_circular_fit_centre_x, 2)2  2.329e+01  7.614e-01  30.585
## poly(crab_circular_fit_centre_y, 2)1  6.391e+00  7.559e-01   8.455
## poly(crab_circular_fit_centre_y, 2)2  1.776e+01  7.726e-01  22.991
## poly(pX, 2)1                      -6.743e+00  7.546e-01  -8.936
## poly(pX, 2)2                      -2.255e+01  7.615e-01 -29.608
## poly(pY, 2)1                      -6.548e+00  7.559e-01  -8.662
## poly(pY, 2)2                      -1.582e+01  7.723e-01 -20.482
## drpLDiff                           4.241e+00  9.919e-02  42.756
## encounters                        -4.566e-03  1.098e-03  -4.159
## drpLDiff:encounters               -1.049e-01  1.826e-02  -5.747
```

```
##
## Correlation matrix not shown by default, as p = 15 > 12.
## Use print(x, correlation=TRUE)  or
##   vcov(x)     if you need it
```

```
## fit warnings:
## Some predictor variables are on very different scales: consider rescaling
```

```
mcp.fnc(df1.4)
```

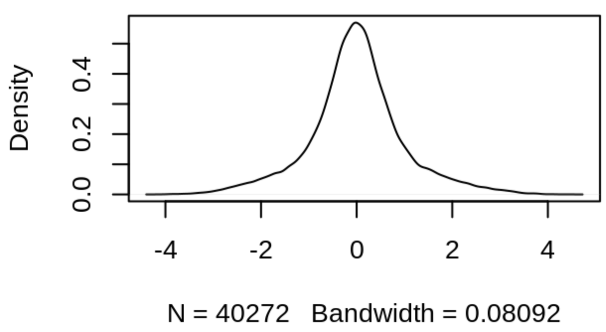

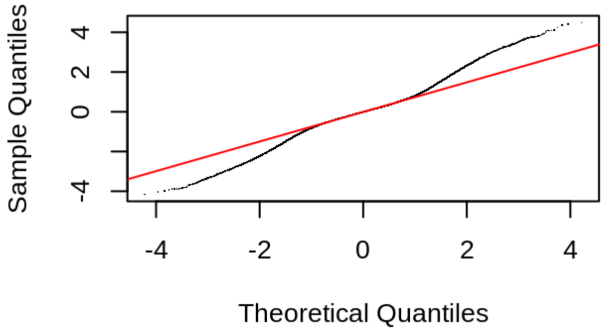

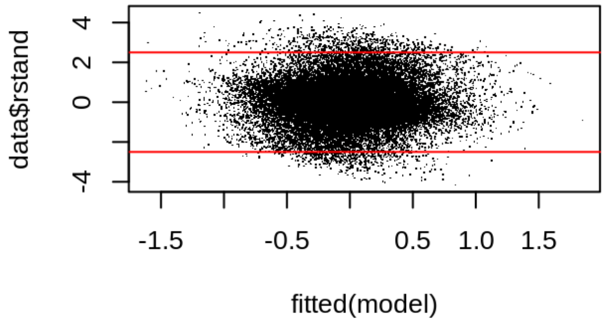


```
# drpLDiff:encounters t= -5.747  p = 9.17e-09 ***

# ggplot(diffData, aes(x=drpLDiff, y=timeDiff, color=factor(encounters), shape=fac
tor(encounters))) + # plot linearity
#   scale_colour_hue(l=40) +
#   geom_smooth(method=lm, se=TRUE)
#


diffData$drpLDiffQ <- quantcut(diffData$drpLDiff, q=2)
nlevels(diffData$drpLDiffQ)
```

```
## [1] 2
```

```
levels(diffData$drpLDiffQ)[1]
```

```
## [1] "[-0.231,0.00034]"
```

```
levels(diffData$drpLDiffQ)[2]
```

```
## [1] "(0.00034,0.251]"
```

```
diffData$drpQe <- ifelse (diffData$drpLDiffQ == levels(diffData$drpLDiffQ)[1], ife
lse(diffData$novelCrab == 1, "Novel|Reduced Disruption", "Repeat|Reduced Disruptio
n"), ifelse(diffData$novelCrab == 1, "Novel|Increased Disruption", "Repeat|Increas
ed Disruption"))
diffData$drpQe <- factor(diffData$drpQe)

ggplot(data=diffData, aes(factor(encounters), timeDiff, color=drpLDiffQ)) +
  geom_boxplot() +
  geom_smooth(method=lm, se=TRUE, aes(group=drpLDiffQ)) +
  labs(y="Capture time compared to previous crab (logged differences)")+
  labs(x="Number of sequential encounters with the same individual crab")+
  theme(panel.grid.major = element_blank(), panel.grid.minor = element_blank(), pa
nel.background = element_blank(), panel.border = element_rect(colour = "black", fi
ll=NA) , legend.position=c(0.8,0.2))
```

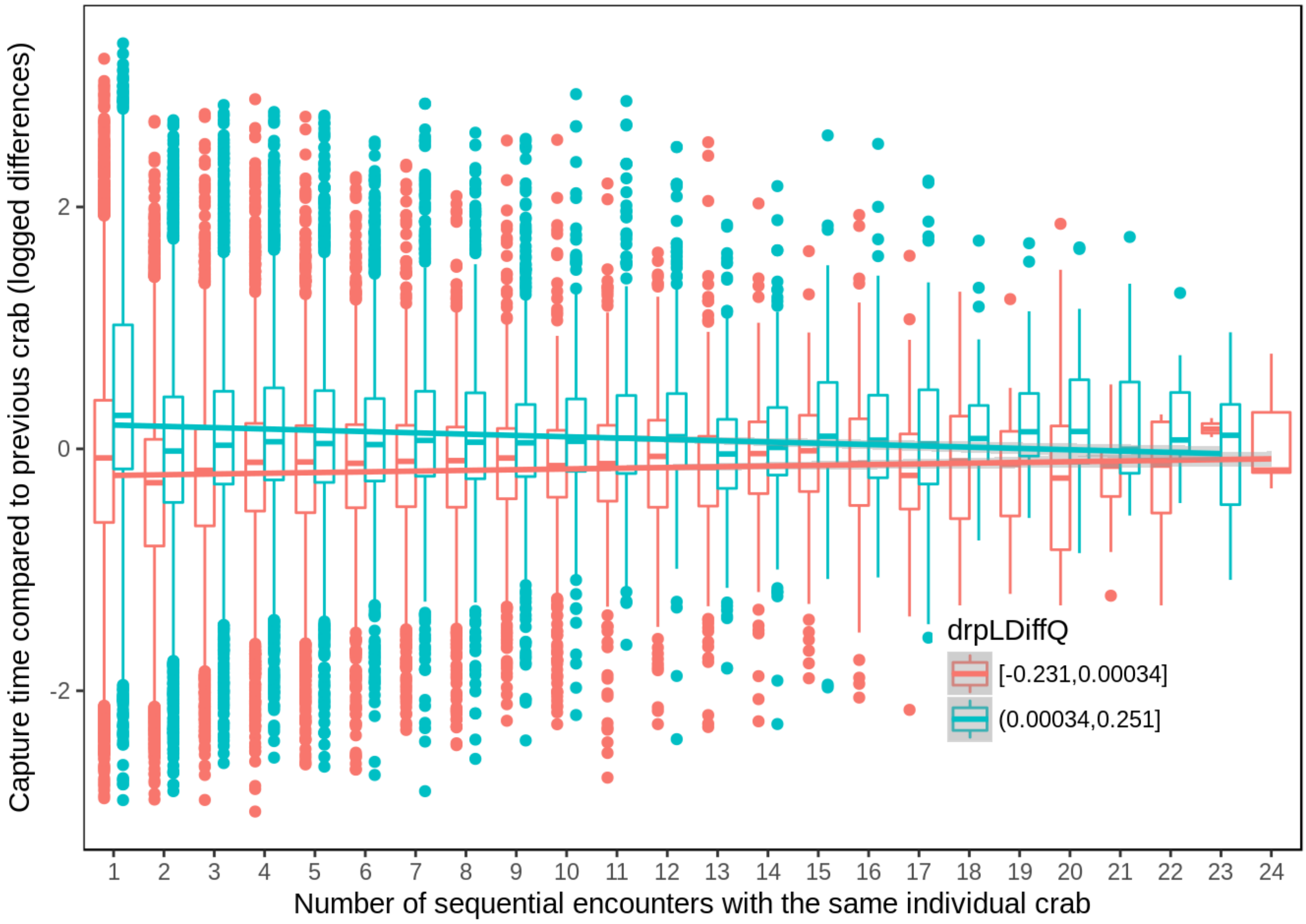

```
p1<-ggplot(diffData, aes(x=drpLDiff, y=timeDiff, color=novelCrab, shape=novelCra
b))+
  scale_colour_hue(l=40) +
  geom_smooth(method=lm, se=TRUE) +
  labs(y="Capture time compared to previous crab (logged differences)")+
  labs(x="Luminance-Disruption compared to previous crab")+
  theme(panel.grid.major = element_blank(), panel.grid.minor = element_blank(), pa
nel.background = element_blank(), panel.border = element_rect(colour = "black", fi
ll=NA) , legend.position=c(0.8,0.2))




df2 <- lmer(timeDiff ~ crab_area + pArea + slide + poly(crab_circular_fit_centre_
x,2) + poly(crab_circular_fit_centre_y,2) + poly(pX,2) + poly(pY,2) +  slide + drp
ADiff*encounters + (1|sessionID), diffData)
summary(df2) #
```

```
## Linear mixed model fit by REML ['lmerMod']
## Formula:
## timeDiff ~ crab_area + pArea + slide + poly(crab_circular_fit_centre_x,
##     2) + poly(crab_circular_fit_centre_y, 2) + poly(pX, 2) +
##     poly(pY, 2) + slide + drpADiff * encounters + (1 | sessionID)
##    Data: diffData
##
## REML criterion at convergence: 94507.9
##
## Scaled residuals:
##     Min      1Q  Median      3Q     Max
## -4.0030 -0.4837 -0.0099  0.4602  4.2992
##
## Random effects:
##  Groups    Name        Variance Std.Dev.
##  sessionID (Intercept) 0.0000   0.0000
##  Residual              0.6108   0.7815
## Number of obs: 40272, groups:  sessionID, 1751
##
## Fixed effects:
##                                    Estimate Std. Error t value
## (Intercept)                      -8.382e-02  1.205e-02  -6.957
## crab_area                        -1.167e-05  4.597e-07 -25.386
## pArea                             1.201e-05  4.588e-07  26.168
## slide                             5.721e-03  6.010e-04   9.520
## poly(crab_circular_fit_centre_x, 2)1  5.707e+00  7.827e-01   7.292
## poly(crab_circular_fit_centre_x, 2)2  2.288e+01  7.892e-01  28.993
## poly(crab_circular_fit_centre_y, 2)1  6.169e+00  7.836e-01   7.873
## poly(crab_circular_fit_centre_y, 2)2  1.742e+01  8.008e-01  21.757
## poly(pX, 2)1                     -5.961e+00  7.827e-01  -7.616
## poly(pX, 2)2                     -2.202e+01  7.893e-01 -27.903
## poly(pY, 2)1                     -6.401e+00  7.836e-01  -8.169
## poly(pY, 2)2                     -1.521e+01  8.005e-01 -19.002
## drpADiff                          1.616e+00  8.419e-02  19.197
## encounters                       -4.445e-03  1.138e-03  -3.906
## drpADiff:encounters              -7.003e-02  1.606e-02  -4.360
```

```
##
## Correlation matrix not shown by default, as p = 15 > 12.
## Use print(x, correlation=TRUE)  or
##   vcov(x)     if you need it
```

```
## fit warnings:
## Some predictor variables are on very different scales: consider rescaling
```

```
# drpADiff:encounters t = -4.360 p = 1.30e-05 ***

p2<-ggplot(diffData, aes(x=drpADiff, y=timeDiff, color=novelCrab, shape=novelCra
b))+
  scale_colour_hue(l=40) +
  geom_smooth(method=lm, se=TRUE) +
  labs(y="Capture time compared to previous crab (logged differences)")+
  labs(x="Chromatic-Disruption compared to previous crab")+
  theme(panel.grid.major = element_blank(), panel.grid.minor = element_blank(), pa
nel.background = element_blank(), panel.border = element_rect(colour = "black", fi
ll=NA) , legend.position=c(0.8,0.2))



df3 <- lmer(timeDiff ~ crab_area + pArea + slide + poly(crab_circular_fit_centre_
x,2) + poly(crab_circular_fit_centre_y,2) + poly(pX,2) + poly(pY,2) +  slide + col
Diff*encounters + (1|sessionID), diffData)
summary(df3)
```

```
## Linear mixed model fit by REML ['lmerMod']
## Formula:
## timeDiff ~ crab_area + pArea + slide + poly(crab_circular_fit_centre_x,
##     2) + poly(crab_circular_fit_centre_y, 2) + poly(pX, 2) +
##     poly(pY, 2) + slide + colDiff * encounters + (1 | sessionID)
##    Data: diffData
##
## REML criterion at convergence: 94693.2
##
## Scaled residuals:
##     Min      1Q  Median      3Q     Max
## -4.0108 -0.4783 -0.0121  0.4612  4.1367
##
## Random effects:
##  Groups    Name        Variance Std.Dev.
##  sessionID (Intercept) 0.0000   0.0000
##  Residual              0.6134   0.7832
## Number of obs: 40272, groups:  sessionID, 1751
##
## Fixed effects:
##                                     Estimate Std. Error t value
## (Intercept)                       -8.313e-02  1.207e-02  -6.885
## crab_area                         -1.199e-05  4.592e-07 -26.115
## pArea                              1.231e-05  4.584e-07  26.844
## slide                              5.674e-03  6.022e-04   9.422
## poly(crab_circular_fit_centre_x, 2)1  6.403e+00  7.838e-01   8.170
## poly(crab_circular_fit_centre_x, 2)2  2.256e+01  7.908e-01  28.530
## poly(crab_circular_fit_centre_y, 2)1  6.503e+00  7.852e-01   8.282
## poly(crab_circular_fit_centre_y, 2)2  1.741e+01  8.025e-01  21.696
## poly(pX, 2)1                      -6.633e+00  7.838e-01  -8.462
## poly(pX, 2)2                      -2.182e+01  7.909e-01 -27.593
## poly(pY, 2)1                      -6.668e+00  7.852e-01  -8.492
## poly(pY, 2)2                      -1.525e+01  8.022e-01 -19.007
## colDiff                           -2.966e-02  1.640e-03 -18.081
## encounters                        -4.372e-03  1.140e-03  -3.834
## colDiff:encounters                 2.048e-03  3.249e-04   6.304
```

```
##
## Correlation matrix not shown by default, as p = 15 > 12.
## Use print(x, correlation=TRUE)  or
##   vcov(x)     if you need it
```

```
## fit warnings:
## Some predictor variables are on very different scales: consider rescaling
```

```
# colDiff:encounters  t= 6.304 p = 2.93e-10 ***

p3<-ggplot(diffData, aes(x=colDiff, y=timeDiff, color=novelCrab, shape=novelCra
b))+
  scale_colour_hue(l=40) +
  geom_smooth(method=lm, se=TRUE) +
  labs(y="Capture time compared to previous crab (logged differences)")+
  labs(x="Crab-Background colour difference compared to previous crab")+
  theme(panel.grid.major = element_blank(), panel.grid.minor = element_blank(), pa
nel.background = element_blank(), panel.border = element_rect(colour = "black", fi
ll=NA) , legend.position=c(0.8,0.9))



df4 <- lmer(timeDiff ~ crab_area + pArea + slide + poly(crab_circular_fit_centre_
x,2) + poly(crab_circular_fit_centre_y,2) + poly(pX,2) + poly(pY,2) +  slide + lum
Diff*encounters + (1|sessionID), diffData)
summary(df4)
```

```
## Linear mixed model fit by REML ['lmerMod']
## Formula:
## timeDiff ~ crab_area + pArea + slide + poly(crab_circular_fit_centre_x,
##     2) + poly(crab_circular_fit_centre_y, 2) + poly(pX, 2) +
##     poly(pY, 2) + slide + lumDiff * encounters + (1 | sessionID)
##    Data: diffData
##
## REML criterion at convergence: 92104.8
##
## Scaled residuals:
##     Min      1Q  Median      3Q     Max
## -4.2947 -0.4999 -0.0157  0.4812  4.4565
##
## Random effects:
##  Groups    Name        Variance Std.Dev.
##  sessionID (Intercept) 0.0000   0.0000
##  Residual              0.5753   0.7585
## Number of obs: 40272, groups:  sessionID, 1751
##
## Fixed effects:
##                                       Estimate Std. Error t value
## (Intercept)                         -8.278e-02  1.169e-02  -7.080
## crab_area                           -1.297e-05  4.335e-07 -29.913
## pArea                                1.325e-05  4.327e-07  30.616
## slide                                5.669e-03  5.832e-04   9.720
## poly(crab_circular_fit_centre_x, 2)1  6.368e+00  7.590e-01   8.390
## poly(crab_circular_fit_centre_x, 2)2  2.305e+01  7.659e-01  30.098
## poly(crab_circular_fit_centre_y, 2)1  6.756e+00  7.604e-01   8.886
## poly(crab_circular_fit_centre_y, 2)2  1.781e+01  7.772e-01  22.912
## poly(pX, 2)1                        -6.595e+00  7.591e-01  -8.689
## poly(pX, 2)2                        -2.237e+01  7.661e-01 -29.207
## poly(pY, 2)1                        -6.958e+00  7.604e-01  -9.149
## poly(pY, 2)2                        -1.556e+01  7.769e-01 -20.026
## lumDiff                             -4.757e-01  1.230e-02 -38.685
## encounters                          -4.288e-03  1.104e-03  -3.883
## lumDiff:encounters                   8.151e-03  2.284e-03   3.569
```

```
##
## Correlation matrix not shown by default, as p = 15 > 12.
## Use print(x, correlation=TRUE)  or
##   vcov(x)     if you need it
```

```
## fit warnings:
## Some predictor variables are on very different scales: consider rescaling
```

```
# lumDiff:encounters t = 3.569 p = 0.000358 ***

p4<-ggplot(diffData, aes(x=lumDiff, y=timeDiff, color=novelCrab, shape=novelCra
b))+
  scale_colour_hue(l=40) +
  geom_smooth(method=lm, se=TRUE) +
  labs(y="Capture time compared to previous crab (logged differences)")+
  labs(x="Crab-Background luminance difference compared to previous crab")+
  theme(panel.grid.major = element_blank(), panel.grid.minor = element_blank(), pa
nel.background = element_blank(), panel.border = element_rect(colour = "black", fi
ll=NA) , legend.position=c(0.8,0.9))



df5 <- lmer(timeDiff ~ crab_area + pArea + slide + poly(crab_circular_fit_centre_
x,2) + poly(crab_circular_fit_centre_y,2) + poly(pX,2) + poly(pY,2) +  slide + pat
Diff*encounters + (1|sessionID), diffData)
summary(df5)
```

```
## Linear mixed model fit by REML ['lmerMod']
## Formula:
## timeDiff ~ crab_area + pArea + slide + poly(crab_circular_fit_centre_x,
##     2) + poly(crab_circular_fit_centre_y, 2) + poly(pX, 2) +
##     poly(pY, 2) + slide + patDiff * encounters + (1 | sessionID)
##    Data: diffData
##
## REML criterion at convergence: 95040.9
##
## Scaled residuals:
##     Min      1Q  Median      3Q     Max
## -3.9199 -0.4770 -0.0122  0.4591  4.2257
##
## Random effects:
##  Groups    Name        Variance Std.Dev.
##  sessionID (Intercept) 0.0000   0.0000
##  Residual              0.6187   0.7866
## Number of obs: 40272, groups:  sessionID, 1751
##
## Fixed effects:
##                                       Estimate Std. Error t value
## (Intercept)                         -8.279e-02  1.213e-02  -6.828
## crab_area                           -1.438e-05  4.509e-07 -31.882
## pArea                                1.470e-05  4.502e-07  32.644
## slide                                5.699e-03  6.048e-04   9.423
## poly(crab_circular_fit_centre_x, 2)1  6.503e+00  7.872e-01   8.260
## poly(crab_circular_fit_centre_x, 2)2  2.251e+01  7.943e-01  28.336
## poly(crab_circular_fit_centre_y, 2)1  6.477e+00  7.885e-01   8.215
## poly(crab_circular_fit_centre_y, 2)2  1.738e+01  8.060e-01  21.557
## poly(pX, 2)1                        -6.697e+00  7.872e-01  -8.507
## poly(pX, 2)2                        -2.174e+01  7.944e-01 -27.362
## poly(pY, 2)1                        -6.656e+00  7.886e-01  -8.441
## poly(pY, 2)2                        -1.516e+01  8.057e-01 -18.817
## patDiff                             -1.240e-02  1.806e-03  -6.865
## encounters                          -4.505e-03  1.145e-03  -3.933
## patDiff:encounters                   4.931e-04  3.367e-04   1.464
```

```
##
## Correlation matrix not shown by default, as p = 15 > 12.
## Use print(x, correlation=TRUE)  or
##   vcov(x)     if you need it
```

```
## fit warnings:
## Some predictor variables are on very different scales: consider rescaling
```

```
# patDiff:encounters  t = 1.464 p = 0.143


grid.arrange(p1,p2,p3,p4, ncol = 2)
```

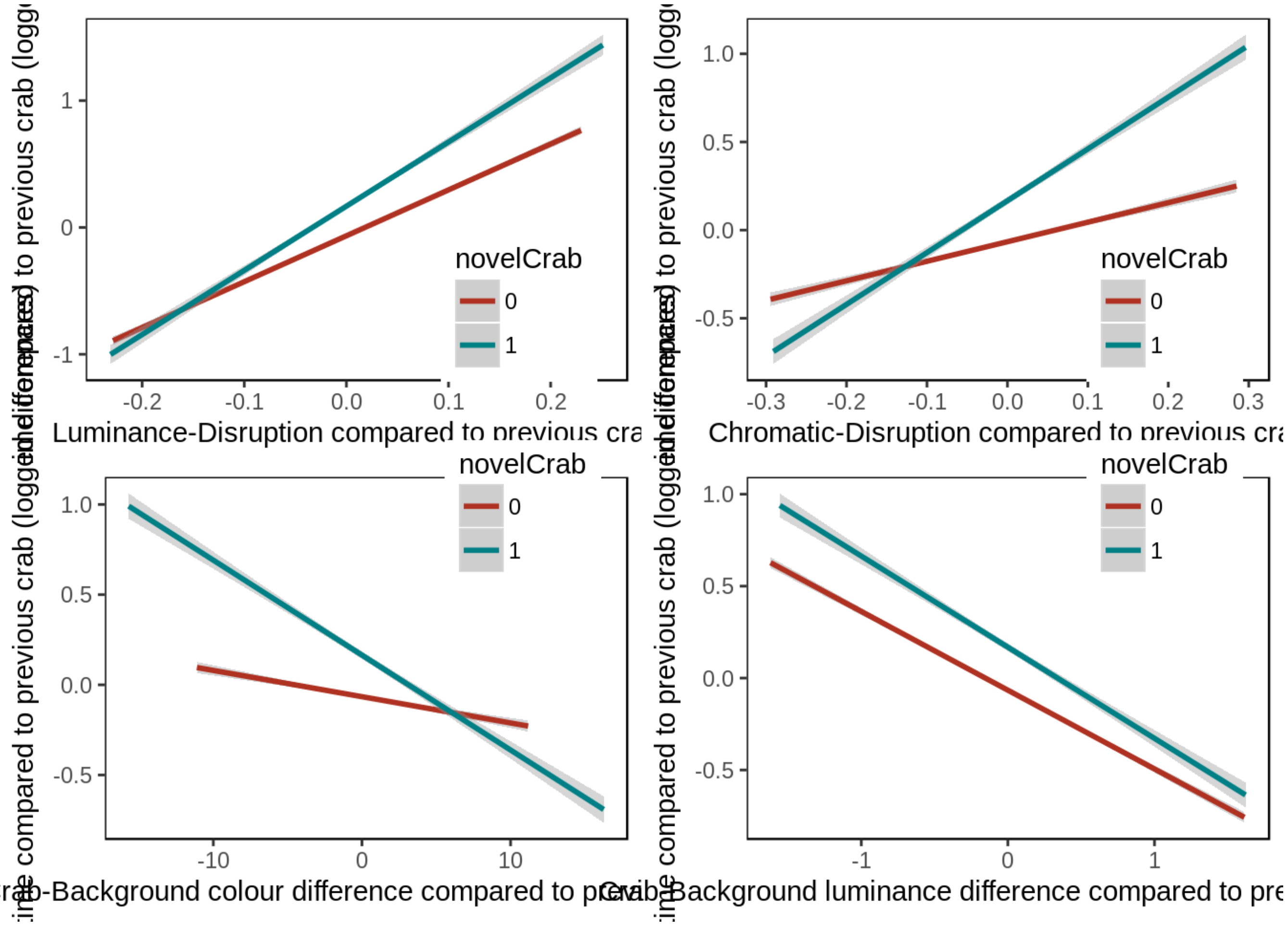


```
# ----------------------------Morph Switching---------------------------

morphData <- subset(diffData, morphSwitch >= 0)
morphData$morphSwitch <- factor(morphData$morphSwitch)

qplot(morphSwitch, timeDiff, novelCrab, data = morphData, geom = "boxplot") + aes
(fill = morphSwitch, novelCrab) +
  theme(panel.grid.major = element_blank(), panel.grid.minor = element_blank(), pa
nel.background = element_blank(), panel.border = element_rect(colour = "black", fi
ll=NA) , legend.position="top")
```

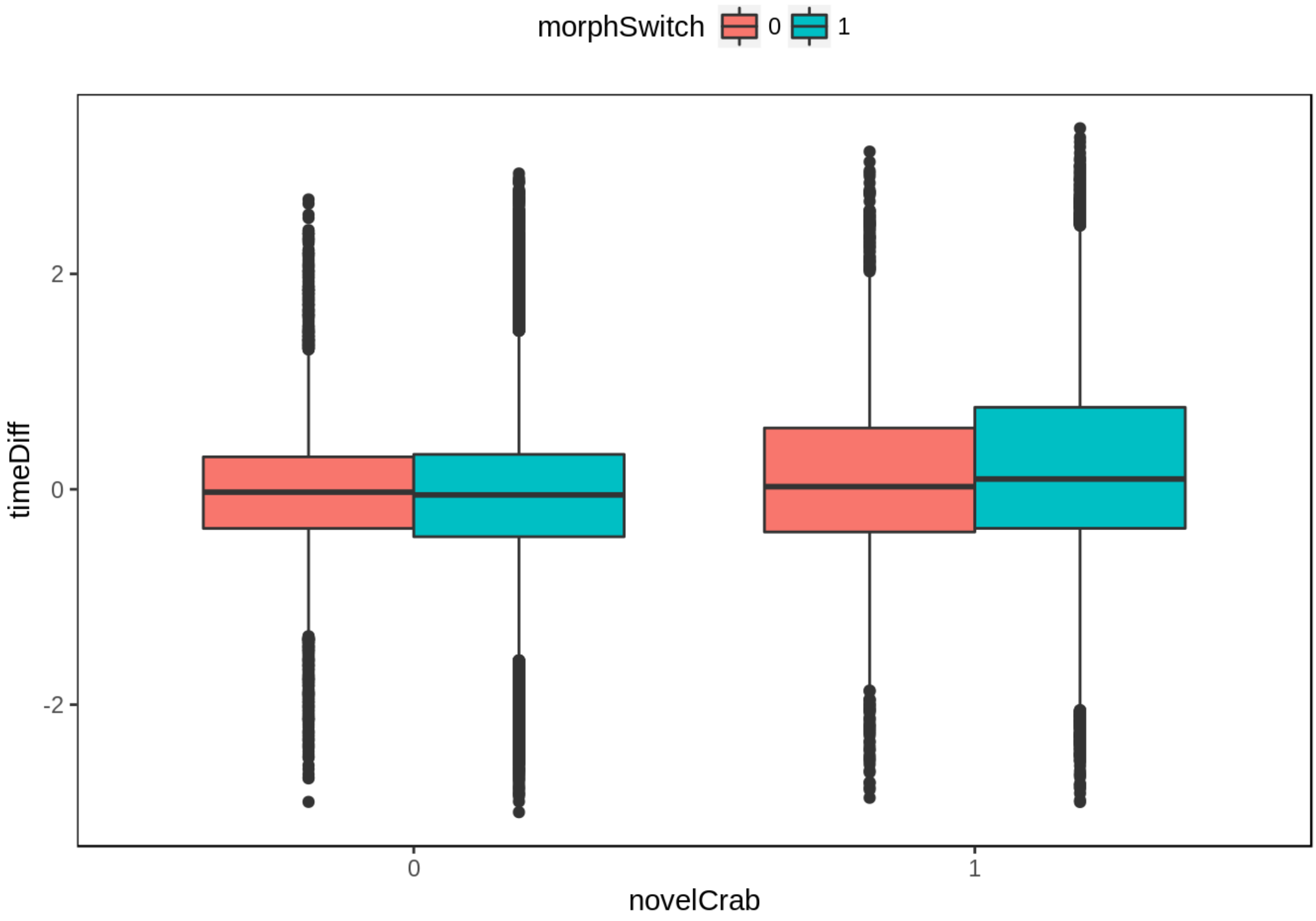


```
mph1 <- lmer(timeDiff ~ crab_area + pArea + slide + poly(crab_circular_fit_centre_
x,2) + poly(crab_circular_fit_centre_y,2) + poly(pX,2) + poly(pY,2) +  slide + mor
phSwitch*encounters + (1|sessionID), morphData)
summary(mph1)
```

```
## Linear mixed model fit by REML ['lmerMod']
## Formula:
## timeDiff ~ crab_area + pArea + slide + poly(crab_circular_fit_centre_x,
##     2) + poly(crab_circular_fit_centre_y, 2) + poly(pX, 2) +
##     poly(pY, 2) + slide + morphSwitch * encounters + (1 | sessionID)
##    Data: morphData
##
## REML criterion at convergence: 79544.7
##
## Scaled residuals:
##     Min      1Q  Median      3Q     Max
## -3.8891 -0.4771 -0.0186  0.4607  4.0659
##
## Random effects:
##  Groups    Name        Variance Std.Dev.
##  sessionID (Intercept) 0.0000   0.0000
##  Residual              0.6409   0.8006
## Number of obs: 33208, groups:  sessionID, 1746
##
## Fixed effects:
##                                     Estimate Std. Error t value
## (Intercept)                       -3.419e-02  2.072e-02  -1.650
## crab_area                         -1.401e-05  4.643e-07 -30.178
## pArea                              1.432e-05  4.633e-07  30.919
## slide                              2.637e-03  7.508e-04   3.512
## poly(crab_circular_fit_centre_x, 2)1  5.441e+00  8.013e-01   6.791
## poly(crab_circular_fit_centre_x, 2)2  2.102e+01  8.079e-01  26.014
## poly(crab_circular_fit_centre_y, 2)1  5.712e+00  8.025e-01   7.117
## poly(crab_circular_fit_centre_y, 2)2  1.552e+01  8.200e-01  18.930
## poly(pX, 2)1                      -5.827e+00  8.013e-01  -7.271
## poly(pX, 2)2                      -1.986e+01  8.080e-01 -24.584
## poly(pY, 2)1                      -6.031e+00  8.027e-01  -7.514
## poly(pY, 2)2                      -1.392e+01  8.198e-01 -16.981
## morphSwitch1                       2.138e-02  1.747e-02   1.224
## encounters                        -4.016e-03  2.974e-03  -1.350
## morphSwitch1:encounters           -4.690e-03  3.313e-03  -1.415
```

```
##
## Correlation matrix not shown by default, as p = 15 > 12.
## Use print(x, correlation=TRUE)  or
##   vcov(x)     if you need it
```

```
## fit warnings:
## Some predictor variables are on very different scales: consider rescaling
```

```
# morphSwitch and interactions are non-significant

mph2 <- lmer(timeDiff ~ crab_area + pArea + slide + poly(crab_circular_fit_centre_
x,2) + poly(crab_circular_fit_centre_y,2) + poly(pX,2) + poly(pY,2) +  slide + mor
phSwitch*novelCrab + (1|sessionID), morphData)
summary(mph2)
```

```
## Linear mixed model fit by REML ['lmerMod']
## Formula:
## timeDiff ~ crab_area + pArea + slide + poly(crab_circular_fit_centre_x,
##     2) + poly(crab_circular_fit_centre_y, 2) + poly(pX, 2) +
##     poly(pY, 2) + slide + morphSwitch * novelCrab + (1 | sessionID)
##    Data: morphData
##
## REML criterion at convergence: 79084.5
##
## Scaled residuals:
##     Min      1Q  Median      3Q     Max
## -4.1181 -0.4840 -0.0056  0.4647  3.9032
##
## Random effects:
##  Groups    Name        Variance Std.Dev.
##  sessionID (Intercept) 0.0000   0.0000
##  Residual              0.6322   0.7951
## Number of obs: 33208, groups:  sessionID, 1746
##
## Fixed effects:
##                                        Estimate Std. Error t value
## (Intercept)                          -9.637e-02  1.861e-02  -5.178
## crab_area                            -1.396e-05  4.611e-07 -30.281
## pArea                                 1.425e-05  4.601e-07  30.980
## slide                                 3.710e-03  7.260e-04   5.110
## poly(crab_circular_fit_centre_x, 2)1  5.457e+00  7.958e-01   6.857
## poly(crab_circular_fit_centre_x, 2)2  2.083e+01  8.024e-01  25.964
## poly(crab_circular_fit_centre_y, 2)1  5.738e+00  7.971e-01   7.198
## poly(crab_circular_fit_centre_y, 2)2  1.545e+01  8.145e-01  18.974
## poly(pX, 2)1                         -6.055e+00  7.959e-01  -7.607
## poly(pX, 2)2                         -1.970e+01  8.025e-01 -24.543
## poly(pY, 2)1                         -6.008e+00  7.972e-01  -7.536
## poly(pY, 2)2                         -1.385e+01  8.143e-01 -17.013
## morphSwitch1                         -2.535e-02  1.269e-02  -1.997
## novelCrab1                            1.302e-01  2.325e-02   5.599
## morphSwitch1:novelCrab1               1.160e-01  2.586e-02   4.485
```

```
##
## Correlation matrix not shown by default, as p = 15 > 12.
## Use print(x, correlation=TRUE)  or
##   vcov(x)     if you need it
```

```
## fit warnings:
## Some predictor variables are on very different scales: consider rescaling
```

```
# significant interaction morphSwitch1:novelCrab1 t= 4.485 p = 7.33e-06 ***
# when swithching to a new crab, swithcing to a differnet morph also increases cap
ture times compared to the previous crab




# create heat map of capture time differences between morphs

morphSwitchData <- subset(diffData, novelCrab == 1)
morphSwitchData$morphCode <- factor(morphSwitchData$morphCode)


sStats <- data.frame(morphCode = levels(morphSwitchData$morphCode))

sStats$mean <- "NA"
sStats$n <- "NA"

# Print off means of each combination of switches
for(i in 1:(length(levels(morphSwitchData$morphCode)))){
  temp <- subset(morphSwitchData, morphCode == levels(morphSwitchData$morphCod
e)[i])
  sStats$mean[i] <- mean(temp$timeDiff)
  sStats$n[i] <- length(temp$timeDiff)
  # print(levels(morphSwitchData$morphCode)[i])
  # print(mean(temp$timeDiff))
}

sStats
```

```
##                  morphCode                mean   n
## 1           black _ black   0.0321056492679919 150
## 2      black _ disruptive    0.462081009178995  96
## 3           black _ green -0.00638934693795831 364
## 4         black _ mottled    0.391401277810897 214
## 5            black _ pale    0.560820769183194 143
## 6         black _ spotted -0.00625622485593078 141
## 7      disruptive _ black    0.100335503513507 115
## 8  disruptive _ disruptive   0.0502198845469607  62
## 9      disruptive _ green   0.0259735581553745 241
## 10   disruptive _ mottled    0.187397511531622 137
## 11      disruptive _ pale     0.24892951898733  97
## 12   disruptive _ spotted    0.223710187975725 100
## 13          green _ black    0.298804605976498 362
## 14     green _ disruptive    0.501545628953969 263
## 15          green _ green   0.0704931245016639 837
## 16        green _ mottled    0.469883099894775 485
## 17           green _ pale    0.535376657522796 340
## 18        green _ spotted    0.278562334932992 344
## 19        mottled _ black  -0.0449383248414005 200
## 20   mottled _ disruptive    0.317565636359212 133
## 21        mottled _ green   -0.116209238938784 466
## 22      mottled _ mottled    0.169043574291937 233
## 23         mottled _ pale    0.179245515954854 176
## 24      mottled _ spotted   -0.108511515590744 196
## 25           pale _ black    -0.05965522076693 131
## 26      pale _ disruptive    0.146718677969962 111
## 27           pale _ green  -0.0870113664365272 331
## 28         pale _ mottled    0.152513465268001 183
## 29            pale _ pale    0.185344483887299 135
## 30         pale _ spotted  -0.0289835203395653 119
## 31        spotted _ black    0.147562091889218 159
## 32   spotted _ disruptive    0.374097279528246 105
## 33        spotted _ green  -0.0143001864029969 333
## 34      spotted _ mottled     0.40897944145526 184
## 35         spotted _ pale    0.281150619953094 133
## 36      spotted _ spotted   0.0920931951749539 123
```

```
# Summary stats used to create heat-map

# save.image()
```
