# Supplementary material for: Variable crab camouflage patterns defeat search image formation
Source: Commun Biol. 2021 Mar 5;4:287. doi: 10.1038/s42003-021-01817-8 (PMC7935895; doi:10.1038/s42003-021-01817-8)
Supplement: Supplementary file 4 — Reporting Summary [file 42003_2021_1817_MOESM4_ESM.pdf]

nature research

Corresponding author(s): Jolyon Troscianko

Last updated by author(s): Dec 7, 2020

Reporting Summary

Nature Research wishes to improve the reproducibility of the work that we publish. This form provides structure for consistency and transparency in reporting. For further information on Nature Research policies, see our Editorial Policies and the Editorial Policy Checklist.

Statistics

For all statistical analyses, confirm that the following items are present in the figure legend, table legend, main text, or Methods section.

n/a	Confirmed	
☐	☒	The exact sample size (*n*) for each experimental group/condition, given as a discrete number and unit of measurement
☐	☒	A statement on whether measurements were taken from distinct samples or whether the same sample was measured repeatedly
☐	☒	The statistical test(s) used AND whether they are one- or two-sided<br>*Only common tests should be described solely by name; describe more complex techniques in the Methods section.*
☐	☒	A description of all covariates tested
☐	☒	A description of any assumptions or corrections, such as tests of normality and adjustment for multiple comparisons
☐	☒	A full description of the statistical parameters including central tendency (e.g. means) or other basic estimates (e.g. regression coefficient) AND variation (e.g. standard deviation) or associated estimates of uncertainty (e.g. confidence intervals)
☐	☒	For null hypothesis testing, the test statistic (e.g. F, t, r) with confidence intervals, effect sizes, degrees of freedom and P value noted<br>*Give P values as exact values whenever suitable.*
☒	☐	For Bayesian analysis, information on the choice of priors and Markov chain Monte Carlo settings
☒	☐	For hierarchical and complex designs, identification of the appropriate level for tests and full reporting of outcomes
☒	☐	Estimates of effect sizes (e.g. Cohen's *d*, Pearson's *r*), indicating how they were calculated

Our web collection on statistics for biologists contains articles on many of the points above.

Software and code

Policy information about availability of computer code

Data collection	The online game used to collect the data was programmed in HTML5 (including HTML, JavaScript, CSS and PHP). This code is public domain.
Data analysis	Image analysis used ImageJ v1.5, and statistics were performed in R v3.4.4. Source code and library versions are included as supplementary material.

For manuscripts utilizing custom algorithms or software that are central to the research but not yet described in published literature, software must be made available to editors and reviewers. We strongly encourage code deposition in a community repository (e.g. GitHub). See the Nature Research guidelines for submitting code & software for further information.

Data

Policy information about availability of data

All manuscripts must include a data availability statement. This statement should provide the following information, where applicable:

- Accession codes, unique identifiers, or web links for publicly available datasets
- A list of figures that have associated raw data
- A description of any restrictions on data availability

The code used to analyse the raw data are provided as an R Markdown document ("Supplementary R Code"), and the raw data are provided as "Supplementary Data".

Field-specific reporting

Please select the one below that is the best fit for your research. If you are not sure, read the appropriate sections before making your selection.

☐ Life sciences ☐ Behavioural & social sciences ☒ Ecological, evolutionary & environmental sciences

For a reference copy of the document with all sections, see nature.com/documents/nr-reporting-summary-flat.pdf

Ecological, evolutionary & environmental sciences study design

All studies must disclose on these points even when the disclosure is negative.

Study description	Human participants played an online "game" where they had to find camouflaged crabs as quickly as possible. The game showed participants crabs in specific sequences so that we could investigate learning effects.
Research sample	Crabs were systematically sampled from natural habitats. Humans were recruited to the game online via social media.
Sampling strategy	Crab photographs were sub-sampled so that each morph type was approximately evenly presented in the dataset. There was no way to control the online game participants, however statistics were used to separate out repeat-players and naive players.
Data collection	Crab photography data were recorded in the field by co-authors. Online data were automatically saved via PHP code.
Timing and spatial scale	The online game went live on 22/12/2016 and data collection was stopped for analysis on 24/05/2018
Data exclusions	Crab images were excluded if the images were out-of-focus, or if the morph type was over-represented. No game data were excluded.
Reproducibility	We provide the raw data and R markdown code for complete transparency and reproducibility.
Randomization	Each game was pseudo-random in design, presenting each participant with a random (non-repeating) background image, and random crab. The likelihood of receiving the same crab in successive slides was 0.8, creating a distribution function of repeat-encounters ideal for our study. Participants could not be controlled (being an online game), however the random nature of the experimental design together with the use of mixed model survival analysis means that differences would be controlled for.
Blinding	Blinding for crab morph categorisation was used.

Did the study involve field work? ☒ Yes ☐ No

Field work, collection and transport

Field conditions	unknown
Location	Falmouth, UK
Access & import/export	No permits required. All crabs were returned to their habitats after photography.
Disturbance	Minimal disturbance to littoral zone species - rocks and seaweed turned over were returned to their original positions.

Reporting for specific materials, systems and methods

We require information from authors about some types of materials, experimental systems and methods used in many studies. Here, indicate whether each material, system or method listed is relevant to your study. If you are not sure if a list item applies to your research, read the appropriate section before selecting a response.

Materials & experimental systems

n/a	Involved in the study	
☒	☐	Antibodies
☒	☐	Eukaryotic cell lines
☒	☐	Palaeontology and archaeology
☐	☒	Animals and other organisms
☐	☒	Human research participants
☒	☐	Clinical data
☒	☐	Dual use research of concern

Methods

n/a	Involved in the study	
☒	☐	ChIP-seq
☒	☐	Flow cytometry
☒	☐	MRI-based neuroimaging

Animals and other organisms

Policy information about studies involving animals; ARRIVE guidelines recommended for reporting animal research

Laboratory animals	*For laboratory animals, report species, strain, sex and age OR state that the study did not involve laboratory animals.*
Wild animals	Wild green shore crabs were collected on beaches around Falmouth. They were kept in buckets of sea water for no more than 1 hour, were photographed, and returned to their habitats.
Field-collected samples	*For laboratory work with field-collected samples, describe all relevant parameters such as housing, maintenance, temperature, photoperiod and end-of-experiment protocol OR state that the study did not involve samples collected from the field.*
Ethics oversight	Ethical approval was given by the University of Exeter ethics board.

Note that full information on the approval of the study protocol must also be provided in the manuscript.

Human research participants

Policy information about studies involving human research participants

Population characteristics	Unknown sample of online (citizen science) participants
Recruitment	Recruited through social media. any bias would have been accounted for by the experimental design.
Ethics oversight	Ethical approval was given by the University of Exeter ethics board.

Note that full information on the approval of the study protocol must also be provided in the manuscript.
